# In silico analysis of the contribution of cardiomyocyte-fibroblast electromechanical interaction to the arrhythmia

**DOI:** 10.3389/fphys.2023.1123609

**Published:** 2023-03-10

**Authors:** Alexander Kursanov, Nathalie A. Balakina-Vikulova, Olga Solovyova, Alexander Panfilov, Leonid B. Katsnelson

**Affiliations:** ^1^ Institute of Immunology and Physiology of the Ural Branch of the Russian Academy of Sciences, Ekaterinburg, Russia; ^2^ Laboratory of Mathematical Modeling in Physiology and Medicine Based on Supercomputers, Ural Federal University, Ekaterinburg, Russia

**Keywords:** mathematical modeling, cardiomyocyte, fibroblasts, cardiac electromechanics, arrhythmia, mechano-electrical feedback, fibroblast-myocyte interaction

## Abstract

Although fibroblasts are about 5–10 times smaller than cardiomyocytes, their number in the ventricle is about twice that of cardiomyocytes. The high density of fibroblasts in myocardial tissue leads to a noticeable effect of their electromechanical interaction with cardiomyocytes on the electrical and mechanical functions of the latter. Our work focuses on the analysis of the mechanisms of spontaneous electrical and mechanical activity of the fibroblast-coupled cardiomyocyte during its calcium overload, which occurs in a variety of pathologies, including acute ischemia. For this study, we developed a mathematical model of the electromechanical interaction between cardiomyocyte and fibroblasts and used it to simulate the impact of overloading cardiomyocytes. In contrast to modeling only the electrical interaction between cardiomyocyte and fibroblasts, the following new features emerge in simulations with the model that accounts for both electrical and mechanical coupling and mechano-electrical feedback loops in the interacting cells. First, the activity of mechanosensitive ion channels in the coupled fibroblasts depolarizes their resting potential. Second, this additional depolarization increases the resting potential of the coupled myocyte, thus augmenting its susceptibility to triggered activity. The triggered activity associated with the cardiomyocyte calcium overload manifests itself in the model either as early afterdepolarizations or as extrasystoles, i.e., extra action potentials and extra contractions. Analysis of the model simulations showed that mechanics contribute significantly to the proarrhythmic effects in the cardiomyocyte overloaded with calcium and coupled with fibroblasts, and that mechano-electrical feedback loops in both the cardiomyocyte and fibroblasts play a key role in this phenomenon.

## 1 Introduction

It is well known that fibrosis is one of the main factors underlying arrhythmia in cardiac tissue (Karagueuzian et al., 2013; Smaill, 2015; Verheule and Schotten, 2021). The contribution of fibrosis to arrhythmogenesis at tissue level is studied very intensively in computational mathematical models (McDowell et al., 2012; Krueger et al., 2014; Nezlobinsky et al., 2021).

To a much lesser extent, mathematical models have analyzed the intracellular mechanisms of the occurrence of arrhythmic cellular dynamics during the interaction between cardiomyocytes and cardiac fibroblasts. The authors of one of the few papers addressing these issues (Sridhar et al., 2017) simulated the electrotonic interaction between cardiomyocytes represented by the *TNNP* model (ten Tusscher et al., 2004) and fibroblasts represented by the *MacCannell* model (MacCannell et al., 2007). They found that an increase in L-type Ca^2+^ current (*i*_*CaL*_) in myocytes combined with depolarization of the resting potential (RP) in fibroblasts (and consequently in myocytes) led to early afterdepolarizations (EADs) of myocytes.

Calcium overload of cardiomyocytes due to an increase in *i*_*CaL*_ is characteristic of a number of pathological situations (Sipido, 2006; Benitah et al., 2010). Therefore, the elucidation of arrhythmogenic factors and mechanisms in such calcium overload seems to be needed for the pathophysiology of the heart.

It is important to note that the depolarization of the RPs in fibroblasts and myocytes, which is considered a necessary factor for the occurrence of triggered activity in the article by Sridhar et al. (2017), did not occur in their simulations as a result of electrical communication of fibroblasts with myocytes and was therefore introduced as an independent additional condition in their model. Meanwhile, there is experimental evidence for a significant depolarizing effect of the cardiomyocyte-fibroblast communication on the RPs (Miragoli et al., 2006). One of the hypotheses proposed and verified in our work is that this potentially arrhythmogenic increase in RP resulting from the interaction between myocytes and fibroblasts arises due to mechanical factors, and that mechano-electrical feedback (MEF) loops in both myocytes and fibroblasts contribute substantially to the triggered activity associated with the increase in *i*_*CaL*_.

Two main circuits of MEF in cardiac cells manifest themselves in health and disease: currents through mechanosensitive ion channels in both cardiomyocytes and cardiac fibroblasts, and MEF mediated by mechanosensitivity of intracellular Ca^2+^ handling in cardiomyocytes (Quinn and Kohl, 2021). The latter involves the mechano-dependence of the kinetics of calcium-troponin complexes (CaTnC), which affects the concentration of intracellular Ca^2+^, and thus, *via* the Na^+^-Ca2+ exchange current, the development of the action potential (AP). Both types of MEF contribute to arrhythmogenesis (Zabel et al., 1996; Kamkin et al., 2000; Katsnelson et al., 2011; Orini et al., 2017).

In this work, our model of electromechanical interaction between a human cardiomyocyte and fibroblasts is used. It includes the MEF in each of these two cell types: the length-dependent dissociation of CaTnC complexes leading to the length dependence of AP in the myocyte, and the mechanosensitive ion channels in fibroblasts (MSC-FB). This model is a further development of our earlier published model describing only the electrical interaction between electromechanically active human cardiomyocyte and cardiac fibroblasts (Bazhutina et al., 2021). The earlier simulation shows that the electrical interaction significantly affects not only the electrical but also the mechanical function of the cardiomyocyte. However, the electrical interaction itself had no effect on the length-dependence of the RP in both myocytes and fibroblasts (Bazhutina et al., 2021).

Now we have added to this model a description of the mechanical interaction between myocyte and fibroblasts and a description of MSC-FB. The description of these channels is based on the approximation to experimental recordings of the voltage-current relationship for such channels obtained by the patch-clamp technique at different fibroblast lengths (Abramochkin et al., 2014).

We use this modified model to assess the possible contribution of MEF in cardiomyocytes and fibroblasts interacting both mechanically and electrically to the triggered activity in cardiomyocytes with increased *i*_*CaL*_ current.

## 2 Material and methods

**FIGURE 1:**
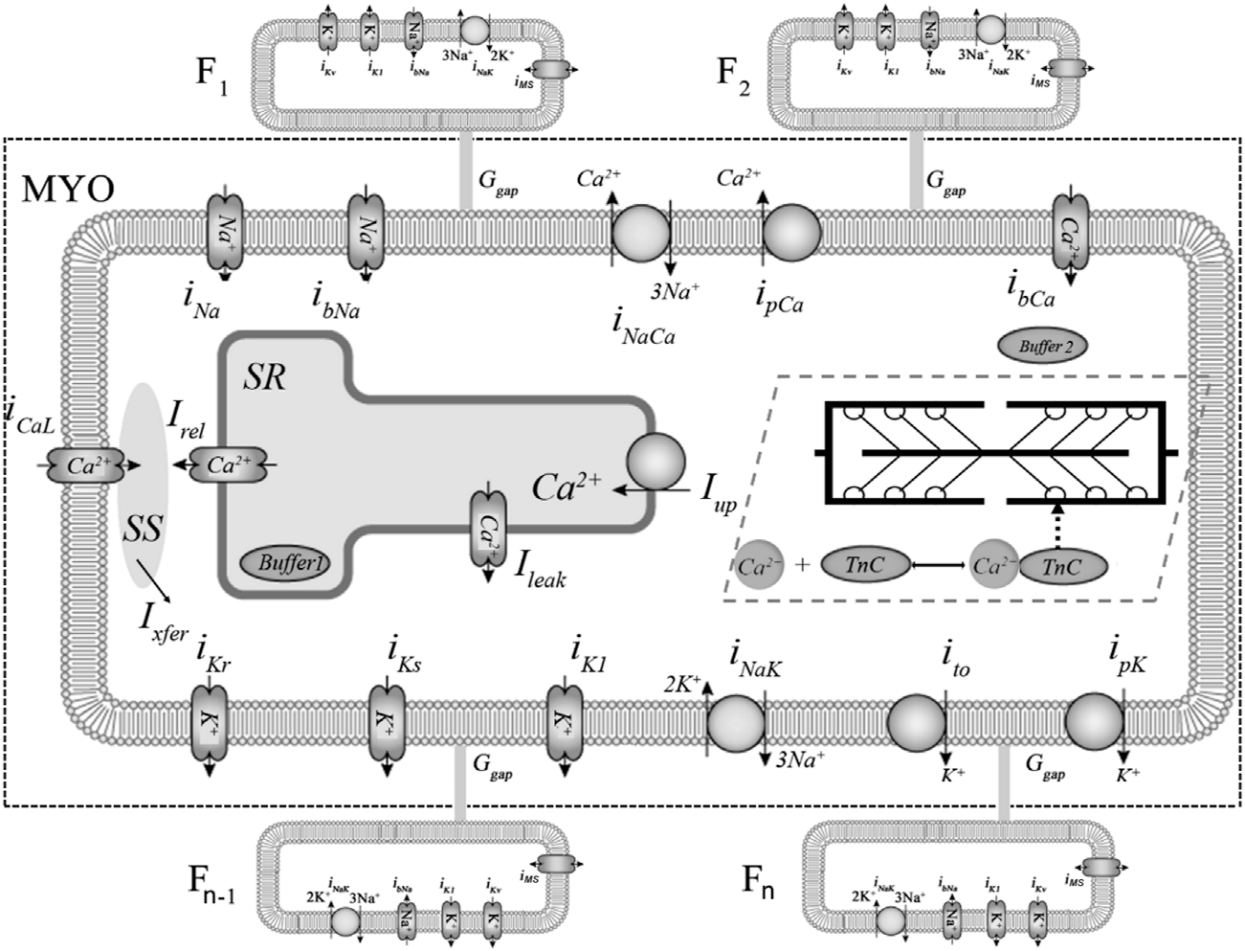
Ionic membrane currents and intracellular calcium homeostasis in the *TP + M* and fibroblast models. The fragment delimited by dashed rectangular box represents the electrophysiological part of the combined *TP + M* model. The fibroblasts are labelled F_1_, F_2_, … , F_n-1_, F_n_. Calcium currents: L-type Ca^2+^ current (*i*_*CaL*_) and background Ca^2+^ current (*i*_*bCa*_). Potassium currents: inward rectifier K^+^ current (*i*_*K1*_); transient outward current (*i*_*to*_); rapid and slow delayed K^+^ rectifier current (*i*_*Kr*_, *i*_*Ks*_); plateau K^+^ current (*i*_*pK*_). Sodium currents: fast Na^+^ current (*i*_*Na*_) and background Na^+^ current (*i*_*bNa*_). Pumps and exchangers: sarcolemmal Ca^2+^ pump current (*i*_*pCa*_); Na^+^-K^+^ pump current (*i*_*NaK*_) and Na^+^-Ca^2+^ exchanger (NCX) current (*i*_*NaCa*_). Calcium translocations: Ca^2+^ release from the sarcoplasmic reticulum (SR) *via* ryanodine receptors into subspace (SS) (*I*_*rel*_); Ca^2+^ diffusion from SS into cytoplasm (*I*_*xfer*_); a small Ca^2+^ leak from into SR into cytoplasm (*I*_*leak*_); Ca^2+^ pumping from the cytoplasm to the SR (*I*_*up*_), where Ca^2+^ is partially buffered (Buffer *1* in SR). Cytoplasmic buffering is divided into two parts: Са^2+^-troponin C complexes (CaTnC) and buffering by other intracellular ligands (Buffer *2*). Non-selective mechanosensitive current in fibroblasts (*i*_*MS*_) was added in *model 2* to account for the mechanical interaction of the myocyte and fibroblasts.

**FIGURE 2:**
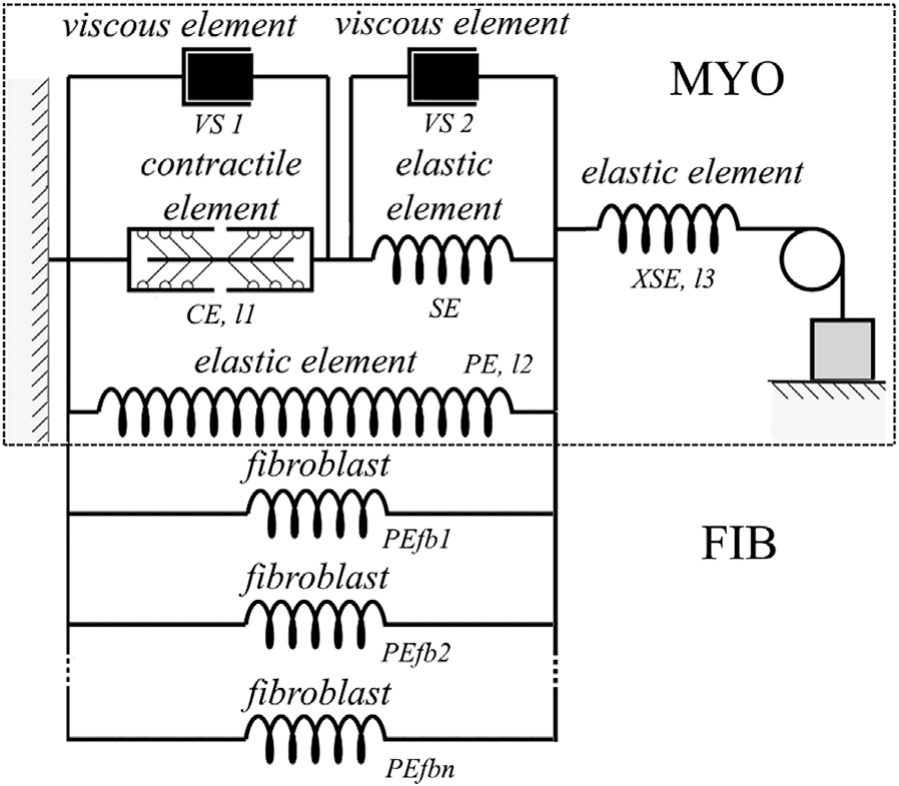
Rheological scheme of the virtual cardiomyocyte described with the *TP + M* model (delimited by dashed rectangular box) linked with the elastic units reflecting fibroblast mechanics. The virtual cardiomyocyte consists of a contractile element generating an active force, three elastic elements, and two viscous elements. The contractile element in the model reproduces the sarcomeres in the cardiomyocyte. Fibroblasts (PEfb1, PEfb2, ..., PEfbn) are attached in parallel to the elastic element (*PE*) of the cardiomyocyte, where *n* is the number of fibroblasts attached.

Here we apply our mathematical model of electromechanical activity for the human cardiomyocyte, the *TP + M* model (Balakina-Vikulova et al., 2020), based on the ten Tusscher-Panfilov model (*TP06*) (ten Tusscher and Panfilov, 2006) and our earlier model of the myocardial mechanical activity and Ca^2+^ handling (Sulman et al., 2008). Figures 1, 2 schematically represent the electrophysiological and mechanical modules of the combined *TP + M* model (dashed boxes). This model simulates both mechanical and electrical behavior of the cardiomyocyte during isometric and afterloaded contractions (Balakina-Vikulova et al., 2020).

The *TP + M* model links electrical and mechanical modules *via* the set of equations for intracellular Ca^2+^ kinetics. In particular, this set includes a thorough description of CaTnC kinetics, as the latter is a key mechanism of the myocardium contraction activation, whereas in the original *TP06* model this kinetics was meant only as an indeterminate part of a generalized intracellular Ca^2+^ buffer. The most important component of the rheological scheme of the *TP + M* model (Figure 2, dashed rectangular boxes) is the contractile element (CE), which represents the sarcomeres in the cardiomyocyte. This element is responsible both for generating the active force developed by the cardiomyocyte and for its shortening due to the cross-bridges (Xbs) formed by the myosin heads attached to the thin actin filaments. Attachment of Xbs during the contractile cycle is regulated by Ca^2+^ ions *via* their binding to troponin C located along the thin filament. An important feature of this model is the mathematical description of cooperativity mechanisms (Sulman et al., 2008) that make the kinetics of CaTnC complexes dependent on the number of attached force-generating Xbs and allow us to simulate and explain a wide range of mechano-calcium and mechanoelectric feedback (MCF and MEF) loops in the cardiomyocyte (Balakina-Vikulova et al., 2020).

Later on we introduced a more detailed description of the ryanodine receptor (RyR) channel gating compared to the original *TP06* model in the improved variant of the *TP + M* model (Bazhutina et al., 2021). This description was taken from the *Shannon* model (Shannon et al., 2004).

For modeling the electrical interaction between fibroblasts and cardiomyocytes, we used the *MacCannell* model (MacCannell et al., 2007), in which the ion currents in fibroblasts were described by separate equations. The differential equation for the transmembrane potential at the *i*-th fibroblast membrane is:

$$\frac{dV_{cfi}}{dt}=-\frac{1}{C_{mf}}\left({i_{cfi}\left(V_{cfi},t\right)+g}_{gap}\left(V_{cfi}-V_{myo}\right)\right),$$ (1)

where $V_{cfi}$ is the membrane potential of the respective fibroblast, $C_{mf}$ is the capacitance of the fibroblast membrane (6.3 pF), $g_{gap}$ is the fibroblast-cardiomyocyte conductance (varied from 0.5 nS to 4.0 nS in simulations), $V_{myo}$ is a membrane potential of the cardiomyocyte, $i_{cfi}$ is the sum of the transmembrane currents of the fibroblasts (Figure 1). In the *MacCannell* model, four membrane ion currents are considered for fibroblasts: a time- and voltage-dependent “delayed-rectifier” K^+^ current *i*_*Kv*_, an inward-rectifying K^+^ current *i*_*K1*_, a Na^+^-K^+^ pump current *i*_*NaK*_, a background Na^+^ “leak” current *i*_*b*Na_. The differential equation for the cardiomyocyte membrane potential is as follows:

$$\frac{dV_{myo}}{dt}=-\frac{1}{C_{myo}}∙({i_{myo}(V_{myo},t)+\overset{n}{\underset{i=1}{\sum }}g_{gap}∙(V}_{myo}-V_{cfi})),$$ (2)

where $C_{myo}$ is myocyte membrane capacitance (185 pF), $i_{myo}$ is the sum of transmembrane currents across the cardiomyocyte membrane from the *TP + M* model, *n* is the total number of fibroblasts. The equation for cardiomyocyte membrane potential is extended compared with the *TP + M* model because of the currents through the fibroblast-cardiomyocyte gap junctions.

Our previously published combined model of the interaction between cardiomyocyte and fibroblasts (Bazhutina et al., 2021) takes into account the mechanical activity of the cardiomyocyte and its MEF but describes only the electrical, not the mechanical, interaction of these cells.

For the new study, we added to the above model a description of the mechanical interaction of the cardiomyocyte with fibroblasts. We considered two factors for this interaction: the presence of a passive elastic force of the fibroblast in response to its deformation, and MSC-FB detected in experiments using patch-clamp technique (Abramochkin et al., 2014). The conductance of these channels and the elastic force depend on the deformation of the fibroblast.

Figure 2 shows a rheological scheme containing fibroblasts as passive elastic elements (PEfb1, PEfb2, … , PEfbn) parallel to the passive elastic element of the cardiomyocyte (PE). Thus, the formula for the force developed by the myocyte (*F*_*myo*_), taking into account the attached fibroblasts, has the following form:

$${F_{myo}=F}_{XSE}=F_{PE}+n∙F_{PEfb}+F_{CE}+F_{VS1},$$ (3)

$$F_{PEfb}=\beta_{2fb}∙\left(e^{\alpha_{2fb}∙l_{2}}-1\right),$$ (4)

where the symbol *F* with each index represents the force developed by the respective element of the rheological scheme (Figure 2); *n* is the number of attached fibroblasts; $\alpha_{2fb}$ and $\beta_{2fb}$ coefficients of the passive elastic force $F_{PEfb}$ developed by the fibroblast PEfb, which are common both for the elastic element PE of the cardiomyocyte and for each fibroblast.

When modeling the mechanosensitive current through the MSC-FB, we relied on the current-voltage characteristics presented by Abramochkin and co-authors for different fibroblast lengths (Abramochkin et al., 2014). We have made linear approximations of their experimental data. Since compression and stretch of the fibroblast changes not only the slope of the I-V curves but also the reversal potential, the formula for the fibroblast mechanosensitive current $\left(i_{MS}\right)$ can be represented as follows:

$$i_{MS}\left(∆l,V_{cfi}\right)=g_{\max}∙a\left(∆l\right)∙\left(V_{cfi}-V_{rev}\left(∆l\right)\right),$$ (5)

where *Δl* is the deformation of the fibroblast during compression and stretch relative to its initial length, *V*_*cfi*_ is the membrane potential of the fibroblast, $g_{\max}$ is the conductance of $i_{MS}$ for the undeformed condition, *a*(*Δl*) is a deformation-dependent factor of $i_{MS}$ (*a(Δl) =* 1 in the undeformed condition), *V*_*rev*_(*Δ* *l*) is the mechanodependent reversal potential.

For a detailed description of the above linear approximation and the selection of parameters of MSC-FB, see the Supplementary Materials to this article.

The mechanosensitive current is accounted for in the sum of ion currents through the fibroblast membrane ( $i_{cfi}$ ) in Eq. 1 above, which describes the transmembrane potential of fibroblasts.

Thus, we have two models for simulating and analyzing the coupling cardiomyocyte with fibroblasts:

- the model of electrotonic interaction between the cells without taking into account mechanical interaction and mechanics of fibroblasts, which has been developed earlier (Bazhutina et al., 2021) (hereinafter referred to as ***model 1***);
- the model that takes into account both electrotonic and mechanical interactions as well as the elasticity of the fibroblast and the mechanosensitive current through the fibroblast membrane (hereinafter referred to as ***model 2***).

*Model 1* is used in this work only to identify, in comparison with it, the features of the mechanical and electrical signals obtained in *model 2* when simulating contractions during normal pacing. This comparison helps to elucidate the contribution of mechanical coupling to the effects of myocyte-fibroblast interaction.

It should be noted that in the context of this study, all fibroblasts are considered homogeneous for simplicity, that is, all values of the parameters of the equations describing each of the *n* fibroblasts coincide. Nevertheless, the model makes it possible to take into account the heterogeneity of fibroblasts interacting with the myocyte in other applications.

A complete list of equations and parameter values of the integrative model of cardiomyocyte-fibroblast electromechanical interaction (*model 2*) used in this study can be found in the Supplementary Materials to this article.

All simulations in this work were performed with a myocyte length of 90% of *L*_*max*_ (where *L*_*max*_ is the length at which the peak active isometric force of the myocyte reaches the maximum value) and a pacing frequency of 1 Hz, unless otherwise noted.

## 3 Results

### 3.1 Contribution of mechanical coupling to the interaction during normal AP development

In this work, we use the term “normal development of AP” (or norm) to denote that no triggered activity (neither extrasystoles nor early/delayed postdepolarizations) occur in cardiomyocytes during their normal pacing.

**FIGURE 3:**
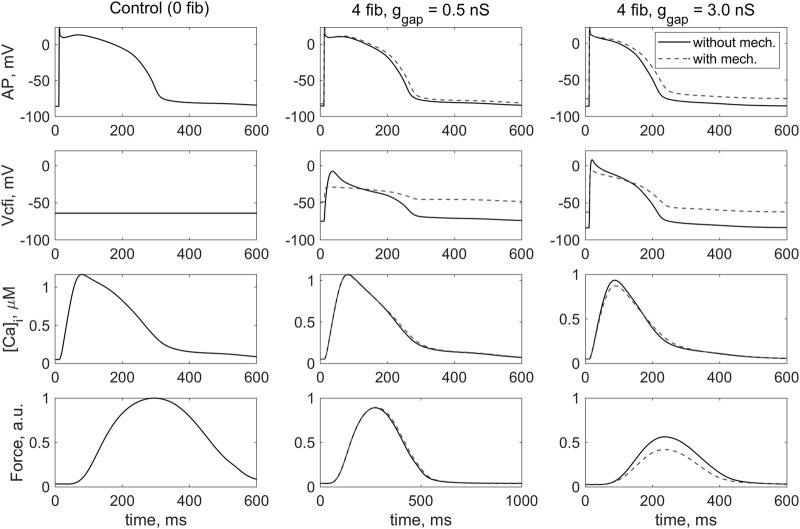
Simulation of the effect of coupled fibroblasts (*n* = 0, 4) and different values of intercellular fibroblast-to-myocyte conductance (*ɡ*_*gap*_ = 0.5, 3.0 nS) in steady-state isometric contractions on the cardiomyocyte action potential (*AP*), the membrane potential across the *i*-th coupled fibroblast (*V*_*cfi*_), the Ca^2+^ transient (*[Ca]_i_*) and the active force generated by the cardiomyocytes (*Force*). The left column contains the results of the simulation of the isometric contraction of the same virtual single cardiomyocyte represented by *TP + M* model. In this case, the membrane potential of the uncoupled fibroblast is shown. The simulation in *model 1* (“*without mech*”) and *model 2* (“*with mech*”) are compared in the middle and left rows. The stimulation frequency is 1 Hz. The force values are normalized to the peak isometric force of the uncoupled myocyte.

Figure 3 shows the results of simulating the isometric contraction of a cardiomyocyte coupled with four fibroblasts in *model 1* and *model 2*. The signals shown were obtained in steady-state conditions at a stimulation frequency of 1 Hz with different conductances of the gap junction (0.5 nS and 3.0 nS).

In our previous study with *model 1*, we have shown that increasing the number of fibroblasts with a gap junction conductance of *ɡ*_*gap*_ = 0.5 nS does not dramatically affect the mechanical and electrical properties of a cardiomyocyte compared with its behavior in isolation (i.e., without connected fibroblasts). Thus, we have assumed that *ɡ*_*gap*_ = 0.5 nS is characteristic for a healthy heart (Bazhutina et al., 2021). Similar effects at *ɡ*_*gap*_ = 0.5 nS are observed for *model 2*. The only significant difference between the response of *model 2* and *model 1* under the same contraction conditions is a completely different configuration of the change in membrane potential of the fibroblast during the interaction as well as the value of this potential at rest (Figure 3, *V*_*cfi*_, middle and right columns). Moreover, both this configuration and the value of fibroblast RP in *model 2* are in better agreement with experimental data (Kohl et al., 2006) than in *model 1*.

In general, it can be seen that when fibroblasts interact with myocyte in *model 2*, which describes in particular the activity of the mechanosensitive current in the fibroblast *i*_*MS*_(*Δl,Vcfi*), the membrane potential of fibroblasts shifts upward and its return to the RP slows when compared with *model 1*, which does not include such ion channels (Figure 3, *V*_*cfi*_, dashed and solid lines). Similar results were obtained even with a smaller number of attached fibroblasts.

We have previously shown in *model 1* (Bazhutina et al., 2021) that an increase in the number of coupled fibroblasts *n* by itself leads to a decrease in action potential duration (APD) in the cardiomyocyte and a decrease in contractile force. In *model 2*, we obtained the same results, although the APD in *model 2* is slightly longer under the same conditions (Figure 3, *AP*).

An increase in the number of attached fibroblasts and an increase in the *ɡ*_*gap*_ leads to a decrease in the force developed by the cardiomyocyte. The force developed in isometric conditions in *models 1* and *2* (Figure 3, *Force,* middle panel) is practically not different in the case of a *ɡ*_*gap*_ of 0.5 nS, which we associate with relative normality (Bazhutina et al., 2021). However, with a larger *ɡ*_*gap*_ equal to 3.0 nS, the isometric force in *model 2* drops even more significantly than in *model 1* where fibroblast mechanics are not accounted for (Figure 3, *Force*, right panel). It is important to note that, as we previously showed (Bazhutina et al., 2021), a high *ɡ*_*gap*_ of 3.0 nS may be associated with severe heart failure, i.e., a pathological state of the myocardium. The simulation in *model 2* suggests that mechanical interaction of the myocyte with fibroblasts exacerbates the failure.

Ion current through MSC-FB in *model 2*, which alters the membrane potential of the fibroblast (Figure 3, *V*_*cfi*_), also has a derived depolarizing effect on the RP of the cardiomyocyte (Figure 3, *AP*). This means that interaction with fibroblasts potentially makes the cardiomyocyte more susceptible to proarrhythmic events. The depolarizing effect of the interaction of the cardiomyocyte with fibroblasts in *model 2* is more pronounced at *ɡ*_*gap*_ = 3.0 nS associated with the pathological state.

**FIGURE 4:**
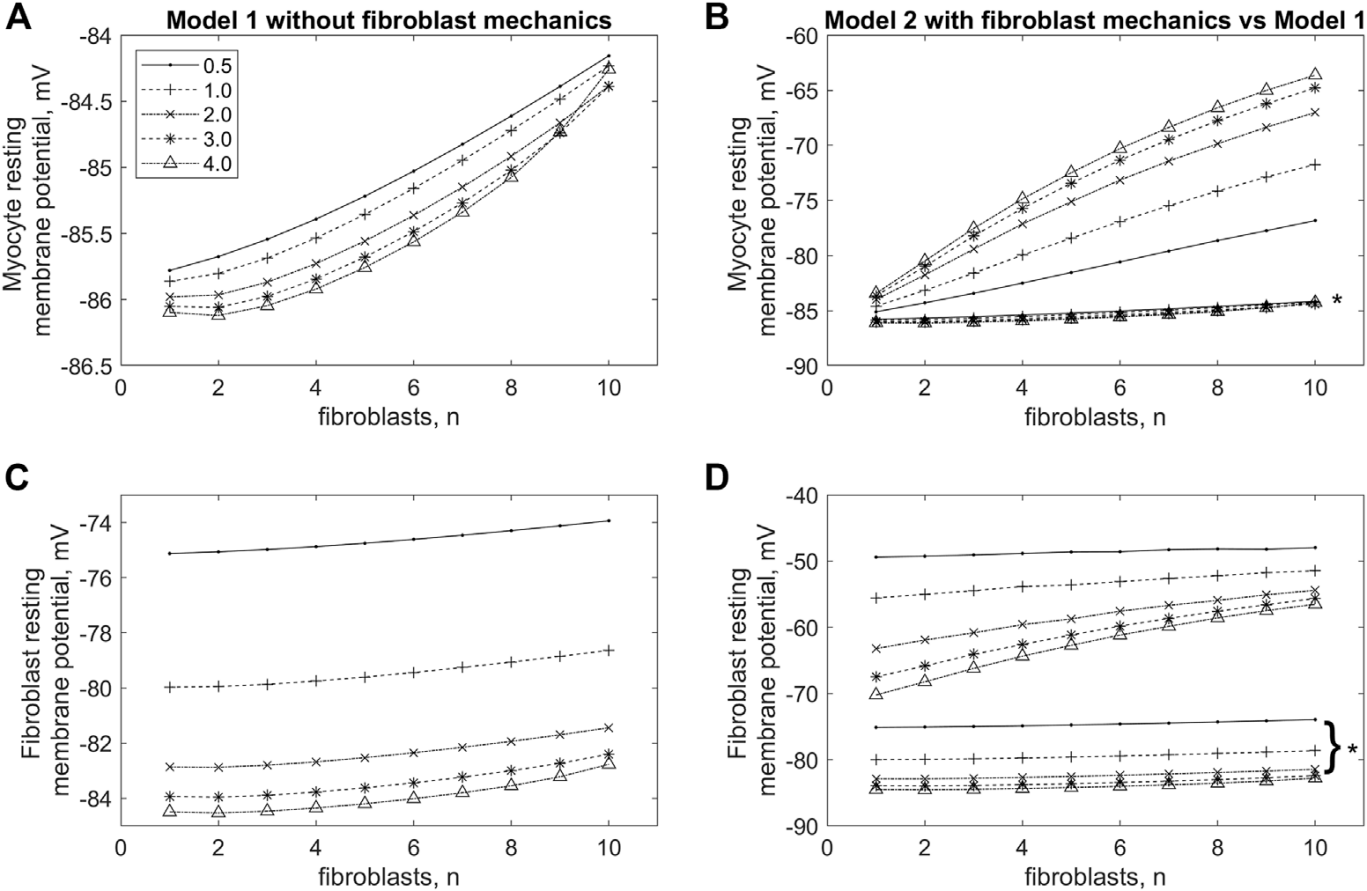
Changes in resting membrane potential in cardiomyocyte **(A,B)** and fibroblasts **(C,D)** in *model 1*, in which the mechano-dependence of fibroblast activity is not considered **(A,C)**, and in *model 2* if compared with *model 1* **(B,D)** with increasing numbers of fibroblasts (*n*) and values of intercellular conductance between fibroblasts and myocytes (*ɡ*_*gap*_). The lines marked with asterisks in panels **(B,D)** are the same as those shown in panels **(A,C)** for *model 1,* but shown at the same scale as the lines for *model 2*.

To investigate this phenomenon in more detail, we compared the RP of cardiomyocyte and fibroblasts in *models 1* and *2* with different numbers of attached fibroblasts *n =* 1, … , 10 and with different gap junction conductance *ɡ*_*gap*_ = 0.5, … , 4.0 nS (Figure 4). In Figure 4B presents dependencies of the cardiomyocyte RP on the number of fibroblasts *n* obtained in *model 2* for a few gap junction conductances *ɡ*_*gap*_. The panel makes it possible to compare all these dependencies not only with each other, but also with the similar curves presented on the same scale and obtained in *model 1* (marked with an asterisk). We see in this panel that RP of the myocyte obtained in *model 1* does not perceptibly depend on either the number of fibroblasts or the gap junction conductance. All these results are in good qualitative agreement with simulations previously performed in a purely electrophysiological *MacCannell* model of the interaction of a cardiomyocyte with fibroblasts (MacCannell et al., 2007). However, the weak dependence of the RP of the cardiomyocyte on the number of fibroblasts is one of the disadvantages of the *MacCannell* model (and accordingly our previous *model 1*). Contrastingly, there is a significant depolarizing contribution of both *n* and *ɡ*_*gap*_ in the cardiomyocyte RP in *model 2*, when *n* or *ɡ*_*gap*_ increases. Another important feature in Figures 4B, D is that all lines representing RPs obtained in *model 1* are significantly lower than all lines for RPs obtained in *model 2*.

Let us pay attention to the main feature of *model 2* compared to *model 1*, which most likely determines RP’s dependencies on *n* and *ɡ*_*gap*_ obtained in simulations on *model 2*. We believe that the significant depolarizing contribution of the $i_{MS}$ current to the RP of fibroblasts (Figure 4D) is just such a feature. Note that there was no $i_{MS}$ current at all in *model 1*. The depolarization of the fibroblast RP inevitably induces the depolarization of the myocyte RP in *model 2* (Figure 4B); moreover, the numerical solutions of the model equations show that the greater *n* and/or *ɡ*_*gap*_, the stronger the induced myocyte RP depolarization. In other words, according to the prediction of *model 2*, the vulnerability of the cardiomyocyte to the triggered activity becomes greater with a pathological growth of the intercellular fibroblast-to-cardiomyocyte conductance and of the number of the neighboring fibroblasts.

Interplay between effects of MCF and MEF in the cardiomyocyte and its electromechanical coupling with fibroblasts equipped with mechanosensitive ion channels is observed when simulating isometric contractions at different initial lengths and different numbers of attached fibroblasts in *model* 2.

**FIGURE 5:**
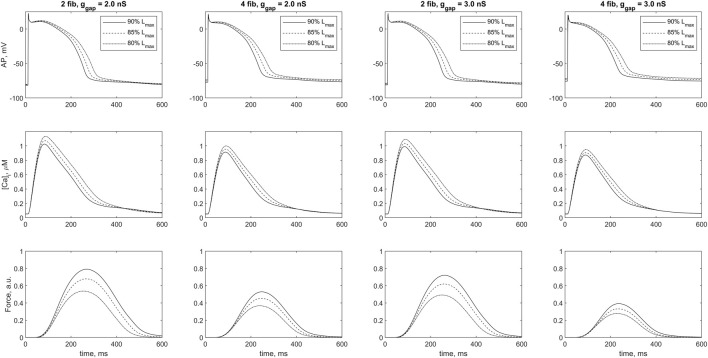
Cardiomyocyte action potential (*AP*), Ca^2+^ transient (*[Ca]_i_*) and active force generated by the cardiomyocyte (*Force*) during a steady-state isometric twitches at different initial lengths in the case of 2 and 4 coupled fibroblasts for intercellular fibroblast-to-myocyte conductances *ɡ*_*gap*_ of 2.0 nS and 3.0 nS in *model 2*. The initial length of cardiomyocyte was set at 80%, 85% and 90% of *L*_*max*_ (where *L*_*max*_ is the length at which the peak active isometric force of the myocyte reaches the maximum value). The stimulation frequency was 1 Hz. Force values are normalized to the peak isometric force of the uncoupled cardiomyocyte.

**FIGURE 6:**
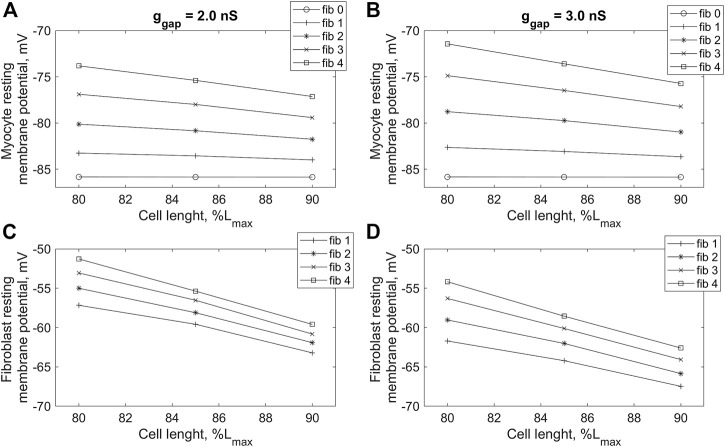
Dependence of resting potential of cardiomyocyte **(A,B)** and fibroblasts **(C,D)** on initial cardiomyocyte length during isometric contractions in *model 2* with different numbers of connected fibroblasts (*n* = 0, … , 4), and gap junction conductances: *ɡ*_*gap*_ = 2.0 nS **(A,C)** and *ɡ*_*gap*_ = 3.0 nS **(B,D)**. The initial cardiomyocyte length was set at 80%, 85% and 90% of *L*_*max*_ (where *L*_*max*_ is the length at which the peak active isometric force of the myocyte reaches the maximum value). The stimulation frequency is 1 Hz.

Figure 5 shows mechanical, electrical, and calcium signals in the cardiomyocyte during its steady-state isometric twitches, when it interacts with 2 and four fibroblasts in *model 2* at two different *ɡ*_*gap*_ values: 2.0 nS and 3.0 nS. In all cases, the initial length of the cardiomyocyte is shown to influence the time course of the AP, the Ca^2+^ transient and the isometric force developed. These changes are based on the mechanisms of MCF and MEF, as we have shown earlier (Balakina-Vikulova et al., 2020). Increasing both *ɡ*_*gap*_ and *n* results in a decrease in peak of Ca^2+^ transients and a significant decrease in peak active isometric force at all lengths. The RP values in cardiomyocyte and fibroblasts are also length-dependent in all cases. A decrease in the initial length of the cardiomyocyte additionally depolarizes the RPs in both the cardiomyocyte and fibroblasts at any number of attached fibroblasts for both tested values of *ɡ*_*gap*_ (Figure 6).

Results of the above part of simulations reveal that complementing electrotonic interaction of the cardiomyocyte and fibroblasts with their mechanical interaction and mechanosensitive currents in the fibroblasts produces noticeable changes in the development of the myocyte isometric force and alters the electrical processes in both the myocyte and the fibroblasts. In particular, the mechanosensitive current in fibroblasts causes a marked depolarization of membrane in rest in both the myocyte and fibroblasts, especially at pathological levels of conductance of the gap junctions between the myocyte and fibroblasts (*ɡ*_*gap*_ ≥ 3.0 nS), and such depolarization is potentially arrhythmogenic. The degree of depolarization of RP depends on both the number of fibroblasts attached and the mechanical conditions of contraction (length). The latter means that RP depolarization is mechano-dependent.

Below in Discussion, we elucidate mechanisms underlying the contribution of MSC-FB to the pronounced differences between fibroblast membrane potentials in *models 1* and *2*, including a significant difference between fibroblast RPs in these models, which in turn leads to differences between cardiomyocyte RPs.

### 3.2 Proarrhythmic contribution of electromechanical interaction between calcium-overloaded cardiomyocyte and fibroblasts

*Model 2* that includes a description of mechano-dependent activity in fibroblasts is used to study the mechanisms of spontaneous electrical activity of cardiomyocytes associated with an increase in L-type calcium current *i*_*CaL*_. Similar study has been performed previously (Sridhar et al., 2017) in mathematical model combining the *TNNP* model (ten Tusscher et al., 2004) with the *MacCannell* fibroblast model (MacCannell et al., 2007). In that work, only the electrotonic interaction between cardiomyocytes and fibroblasts was considered, and the mechanics for both types of interacting cells were not taken into account. The authors used experimentally confirmed effect of significant depolarization of RP in the communicating fibroblasts and cardiomyocytes with an increase in the number of fibroblasts (Miragoli et al., 2006). In their model, however, such significant effect did not occur by itself due to only electrotonic intercellular interaction. Therefore, they set it as an additional external condition. Under this external condition, an increase in gap junction conductance together with an increase in *i*_*CaL*_ was shown to increase vulnerability to the triggered activities manifesting themselves as EADs (Sridhar et al., 2017).

**FIGURE 7:**
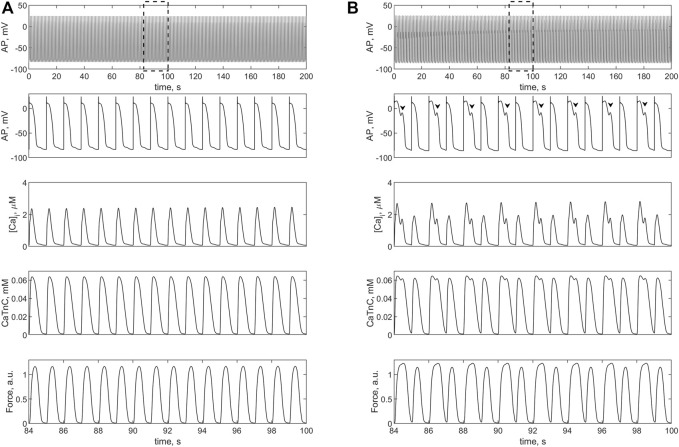
Isometric contractions of a cardiomyocyte at a length of 90%*L*_*max*_ in the case of a ***1.4-fold*** increase (compared to the norm) in L-type calcium current conductance. **(A)** simulation in the *TP + M* model (i.e., without fibroblasts), where no triggered activity occurs. **(B)** simulation in *model 2* with electromechanical interaction with four fibroblasts at *ɡ*_*gap*_ = 3.0 nS. Upper panels: cardiomyocyte APs in the time interval from 0 to 200 s at a pacing rate of 1 Hz. Other panels: details of APs, Ca^2+^ transients (*[Ca]_i_*), concentration of calcium-troponin complexes (*CaTnC*) and isometric force signals (*Force*) in the interval from 84 to 100 s, marked by dashed rectangles in the upper panels. The EADs in *model 2* are shown by arrows.

**FIGURE 8:**
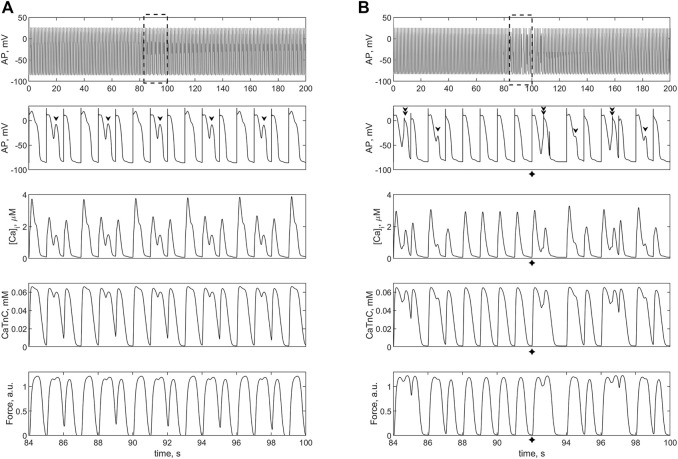
Isometric contractions of a cardiomyocyte at a length of 90%*L*_*max*_ in case of a ***twofold*** increase (compared to the norm) of the L-type calcium current conductance. **(A)** simulation in the *TP + M* model (i.e., without fibroblasts). Proarrhythmic disturbances in the isolated cardiomyocyte occur in the form of EADs (shown by arrows), not extrasystoles. **(B)** simulation in *model 2* with electromechanical interaction with 2 fibroblasts at *ɡ*_*gap*_ = 3.0 nS. Extrasystoles (shown by crosses) start at 92 s. Upper panels: cardiomyocyte APs in the time interval from 0 to 200 s at a pacing rate of 1 Hz. Other panels: details of APs, Ca^2+^ transients (*[Ca]_i_*), concentration of calcium-troponin complexes (*CaTnC*) and isometric force signals (*Force*) in the interval from 84 to 100 s marked by dashed rectangles in the upper panels.

**FIGURE 9:**
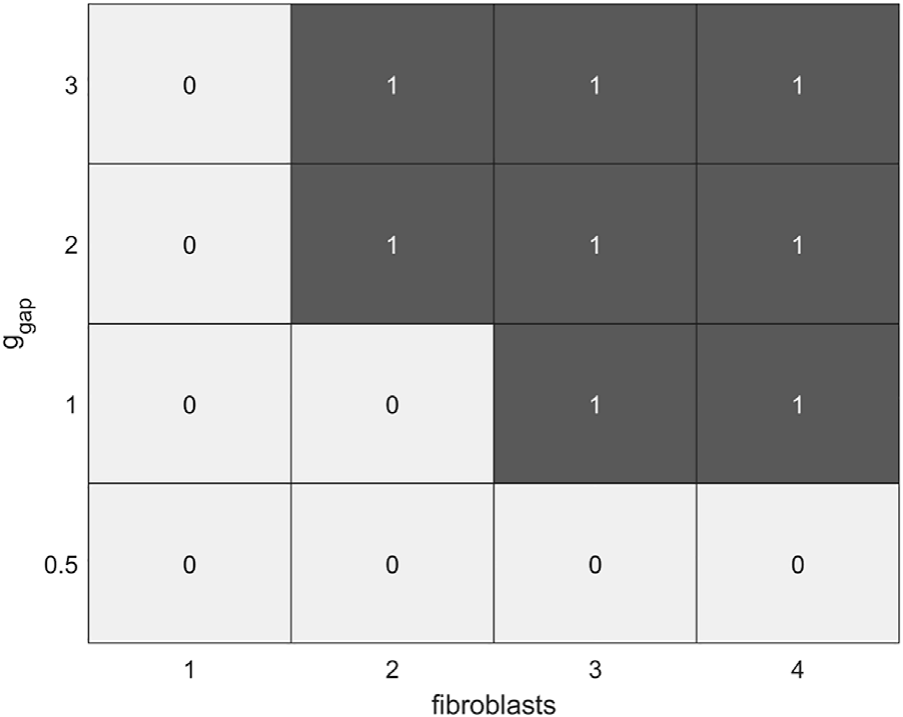
Diagram of the dependence of the occurrence of EADs (denoted by 0) and extrasystoles (denoted by 1) in the calcium-overloaded cardiomyocyte (twofold increase in *i*_*CaL*_ compared to the norm) in *model 2* on a number of fibroblasts (*n* = 0, … , 4) and intercellular fibroblast-to-myocyte conductances (*ɡ*_*gap*_ = 0.5, 1.0, 2.0, 3.0 nS) during isometric contractions at an initial cardiomyocyte length of 90%*L*_*max*_.

In this work, the same protocol of increasing *i*_*CaL*_ to achieve arrhythmic cellular dynamics in the cardiomyocyte was repeated in *model 2* involving electromechanical interaction between the cardiomyocyte and the fibroblasts attached to it. However, in contrast to the cited work (Sridhar et al., 2017), we do not introduce depolarization of RPs in fibroblasts as an additional external condition for the numerical experiment. This depolarization in *model* 2 occurs by itself, as shown above. As shown in Figures 7–9, in *model* 2 we obtained not only EADs but even early extrasystoles in conditions of increased *i*_*CaL*_.

Figure 7 shows the simulation results with 1.4-fold increase in the conductance of *i*_*CaL*_ compared to the normal conductance of 5e-5 (F⋅s)^−1^ (see Supplementary Materials). This change in *i*_*CaL*_ in the same single cardiomyocyte does not cause triggered activity (Figure 7A), but in the case of its electromechanical interaction with four fibroblasts EADs occurs in it (Figure 7B). Note that the 1.4-fold increase is smaller than twofold increase in this current implemented by Sridhar et al. to obtain EADs (Sridhar et al., 2017).

Then we applied the twofold increase in *i*_*CaL*_ in the *TP + M* model of the single cardiomyocyte and in *model 2* (Figure 8). In the electromechanical *TP + M* model that forms the basis for *model 2*, the twofold increase in *i*_*CaL*_ in the single cardiomyocyte gradually (twitch-by-twitch) causes calcium overload, which in turn leads to the development of EADs (Figure 8A). In other words, in the electromechanical *TP + M* model (as opposed to the electrical *TNNP* model used by Sridhar et al.), EADs occur when the cardiomyocyte is overloaded with calcium due to twofold increase in *i*_*CaL*_ even without attachment of fibroblasts. The mechanism of these triggered activities, particularly the role of MCF and MEF loops in the myocyte, is discussed below (see Discussion). In the ensemble with fibroblasts in *model 2*, the twofold increase in *i*_*CaL*_ results not only in EADs but also in early extrasystoles manifesting themselves as both extra APs and extra contractions (Figure 8B). For early extrasystoles to occur in the cardiomyocyte after twofold increase in *i*_*CaL*_, it turned out to be sufficient to attach only two fibroblasts to the cardiomyocyte rather than four. Note that early ventricular extrasystoles have been demonstrated in a patient in a clinical trial, such as in Figures 9C, D in the article by Shabetai et al. (1968).

As mentioned above, the presence of MSC-FB leads to depolarization of cardiomyocyte RP. This depolarization increases with an increase in *ɡ*_*gap*_ and in the number of fibroblasts and thus contributes to the proarrhythmic vulnerability of the myocyte. In particular, when cardiomyocytes are overloaded with calcium, MSC-FB may enhance triggered activities in such cardiomyocytes up to extrasystoles (compare Figures 8A, B).

Thus, in *model 2*, interaction with fibroblasts of cardiomyocytes overloaded with calcium contributes to the occurrence of both EADs and extrasystoles in these cells. The latter occur at sufficiently large *ɡ*_*gap*_ values. The diagram in Figure 9 shows that cardiomyocyte vulnerability to both types of the triggered activities in *model 2* increases with an increase in the number of attached fibroblasts and/or an increase in gap junction conductance *ɡ*_*gap*_. The more fibroblasts attached, the lower the threshold of *ɡ*_*gap*_ at which the corresponding disorders occur. Variations of other parameters including the myocyte lengths do not change significantly the overall picture. For example, when a cardiomyocyte interacts with 2 fibroblasts at *ɡ*_*gap*_ = 2 nS and initial length of 80% of *L*_*max*_, no extrasystoles occur, only EADs.

Figures showing the simulation of triggered activity with an increase in the conductance of *i*_*CaL*_ in *model 1*, in which only the electrotonic interaction of the cardiomyocyte with fibroblasts is considered, are not shown here. Arrhythmias in *model 1* are identical to those in the *TP + M* model, which reproduces the electromechanical behavior of the single cardiomyocyte. Only EADs do arise in *model 1*, and this occurs at the same and only at the same values of the *i*_*CaL*_ conductance as in the simulated cardiomyocyte without fibroblasts attached.

## 4 Discussion

### 4.1 MEF in the model of cardiomyocyte electrically connected with fibroblasts

There are a number of experimental data sets indicating that communication between cardiomyocytes and fibroblasts may itself contribute to arrhythmogenesis (Yue et al., 2011; Rohr, 2012; Vasquez and Morley, 2012; McArthur et al., 2015; Pellman et al., 2016; Sanchez et al., 2019; Hall et al., 2021; Nicin et al., 2022). However, our understanding of the mechanisms underlying this pathological manifestation of cardiomyocyte-fibroblast interaction is still incomplete. In the present paper, we apply mathematical modeling to uncover such possible mechanisms associated with the electromechanical coupling of human cardiomyocytes and fibroblasts.

To study the effect of fibroblasts on the electrical and mechanical activity of human myocardium, we have previously constructed a model of the electrical interaction of a human cardiomyocyte with fibroblasts (Bazhutina et al., 2021), which we refer to here as *model 1*. *Model 1* is an extension of the *MacCannell* model (MacCannell et al., 2007). As is well known, the cardiomyocyte in the *MacCannell* model is represented by the electrical *TNNP* model (ten Tusscher et al., 2004), whereas in *model 1* we replaced the *TNNP* model with our electromechanical *TP + M* model that combines the *TP06* model (a further development of the *TNNP* model) and a module for simulation of cardiomyocyte mechanics (Balakina-Vikulova et al., 2020). This allowed us to analyze some aspects not only the electrical but also the mechanical response of a cardiomyocyte to electrical interaction with fibroblasts in *model 1*. In addition, MEF and MCF, mediated by Xbs and CaTnC kinetics included in the *TP + M* model, allowed us to evaluate the effects of the mechanical pattern of cardiomyocyte contractions on its electrical function in an ensemble with fibroblasts. At the same time, there was no actual mechanical interaction between the myocyte and fibroblasts in *model 1*.

Some of the results obtained in *model 1* are shown here (Figure 3) in comparison with similar simulations performed in *model 2*. A wide range of numerical experiments performed in *model 1* and their detailed analysis were published by us earlier (Bazhutina et al., 2021). A brief summary of that previous work is as follows.

Specifically, in that study, we showed that the isometric force of the myocyte did not decrease by more than 20% when two fibroblasts electrically interacted with this myocyte with a conductance of ∼0.5 nS, as compared to the same simulated single cardiomyocyte. Furthermore, at a *ɡ*_*gap*_ of 0.5 nS, both the slope of the isometric “Length-Force” relationship and the effect of load-dependent relaxation during isotonic twitches were found to be nearly the same for the cardiomyocyte interacting with two fibroblasts and for the single cardiomyocyte. As the number of attached fibroblasts continues to increase, the isometric force of the myocyte decreases sharply at the same *ɡ*_*gap*_ of 0.5 nS. However, a much more substantial decrease in force even in the case of only two fibroblasts, occurred at higher simulated junctional conductance values (when the range of *ɡ*_*gap*_ from the (MacCannell et al., 2007) article was tested), especially at the *ɡ*_*gap*_ of 3.0 nS. This decrease was accompanied by a significant decrease in the peak value and duration of the Ca^2+^ transient, the slope of the “Length- Force” relationship, and the almost complete disappearance of the effect of load dependence of relaxation during isotonic twitches. This suggests that the *ɡ*_*gap*_ of 3.0 nS is a feature of severe pathologies. There is ample experimental evidence for the increase in gap junction conductance associated with the expression of connexins Cx45 and Cx43 in various pathological conditions (Doble and Kardami, 1995; Camelliti et al., 2004; Miragoli et al., 2006; Vasquez et al., 2010; McArthur et al., 2015; Pecoraro et al., 2015). For example, a significant increase in Cx43 levels was found in fibroblasts after myocardial infarction in rats (Miragoli et al., 2006; Vasquez et al., 2010; McArthur et al., 2015), suggesting that increased Cx43 expression in fibroblasts from hearts exposed to infarction may lead to increased formation of gap junctions between myocytes and fibroblasts. These findings have allowed McArthur et al. (2015) to suggest that increased electrical coupling between myocytes and fibroblasts in the context of cardiac disease may affect myocardial electrical activity and contribute to the occurrence of arrhythmias. Our previously performed simulations (Bazhutina et al., 2021) give reasons to believe that such pathological conditions not only affect electrical activity but also lead to a dramatic decrease in cardiomyocyte mechanical activity and weak MCF- and MEF-loops associated with CaTnC kinetics in the cardiomyocyte. The greater the number of attached fibroblasts and especially the conductance of gap junctions, the weaker both feedback loops.

### 4.2 Resting membrane potential in cardiomyocyte and fibroblasts

Despite all the above findings, *model 1* accounting for only the electrical communication of the cardiomyocyte with fibroblasts, failed to simulate experimentally observed significant depolarization of the RP in the myocyte and fibroblasts communicating with it in response to an increase in their number (Miragoli et al., 2006) and increased vulnerability to triggered activity (e.g., EADs and/or extrasystoles) of the cardiomyocyte due to this communication in pathological conditions.

A significant effect of myocyte-fibroblast communication on RPs occurs in *model 2*, as shown in Figures 3–6. This effect is primarily due to the significant depolarization of the fibroblast RP in *model 2* compared to *model 1*. The differences between the membrane potentials in *model 2* and *model 1* can be clearly seen in Figure 3. The contribution of the MSC-FB included in *model 2* to these differences is discussed further.

**FIGURE 10:**
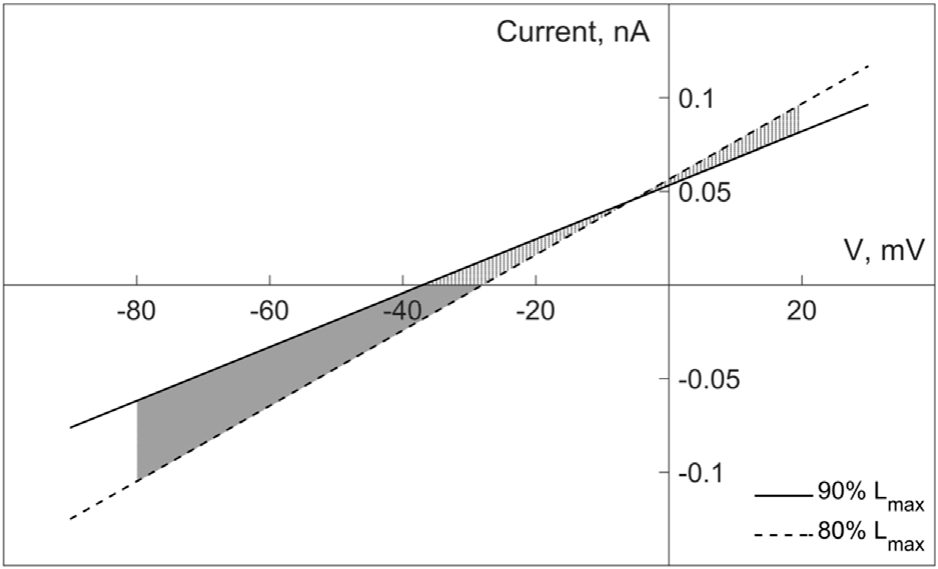
Current-voltage relationships for the non-selective mechanosensitive current in fibroblasts in *model 2*, given for two lengths of 80% and 90% of *L*_*max*_ (where *L*_*max*_ of fibroblasts is related to *L*_*max*_ of cardiomyocyte). The two parts of the shaded area correspond to the different polarity of the mechanosensitive current in the fibroblast, determining its depolarizing (the lower part) and repolarizing (the upper part) contribution to the fibroblast membrane potential for any instant length of the fibroblast within the range of 80%–90% of *L*_*max*_.

To do this, refer to Figure 10 that shows two length-dependent voltage-current relationships for MSC-FB in *model 2*, which experimental justification and numerical interpolation of the corresponding experimental relationship are presented in more detail in the Supplementary Materials (Section “III. Fibroblasts—Myocyte Electromechanical Interaction”). In particular, the formulation of the fibroblast mechanosensitive current $\left(i_{MS}\right)$ contains a length-dependence of the reversal potential and conductance. The solid line in Figure 10 is drawn for a constant cardiomyocyte length of 90% of *L*_*max*_ and the dashed line for 80% of *L*_*max*_, where *L*_*max*_ is the length at which the myocyte develops maximum isometric force (*L*_*max*_, in terms of the sarcomere lengths, corresponds to 2.23 µm). All contractions shown in Figures 3–9 begin at the initial length of 90% of *L*_*max*_. Note that although in this work we are simulating isometric contractions, this is isometry of the entire virtual preparation presented in Figure 2. The preparation has a certain internal compliance (elastic elements XSE and SE) (Sulman et al., 2008) so that the sarcomeres shorten during contractions. Nevertheless, their length remains higher than 80% of *L*_*max*_. Along with the cardiomyocyte, the fibroblast parallel to it also remains in the range between 80% and 90% of its *L*_*max*_ (here the *L*_*max*_ of the fibroblast is associated with the *L*_*max*_ of the cardiomyocyte). Therefore, the instant voltage-current relationship for MSC-FB in the range of possible lengths lies within the shaded area between the solid and dashed lines in Figure 10.

According to Eq. 1, the current through the non-selective MSC-FB in *model 2* is an additional term to the sum of the ionic currents through the selective channels considered in both *model 2* and *model 1*. The contribution of this term to the dynamics of the fibroblast membrane potential can be either depolarizing or repolarizing, depending on the direction of the current through the MSC-FB: a negative current makes a depolarizing contribution, a positive one a repolarizing one.

Based on these circumstances, we compare the dynamics of the membrane potentials of fibroblasts in *model 2* and *model 1* in Figure 3. For certainty, we consider the potentials in the middle panel in the second row (of course, the same comparison is also correct for the right panel). After a stimulus, the fibroblast membrane potential lies in the lower part of the shaded area between the length-dependent voltage-current relationships (Figure 10) and is guaranteed to remain there at least as long as its value is no higher than −38 mV. It returns to the same subarea during the repolarization phase as soon as it falls below −38 mV. Since the current through MSC-FB is negative in this subarea, its contribution to the formation of the fibroblast membrane potential turns out to be depolarizing, thus underlying the depolarization of the fibroblast RP in *model 2* compared to *model 1* in Figure 3.

If, on the other hand, the fibroblast potential is above −30 mV during its development (see the same panel in Figure 3), it enters the upper part of the shaded area in the voltage-current diagram (Figure 10). Therefore, the current through the MSC-FB becomes positive, and its contribution to the formation of the fibroblast membrane potential becomes repolarizing. This explains the decrease in the spike and plateau of the fibroblast membrane potential in *model 2* compared to *model 1* (Figure 3).

Depolarization of fibroblast RP inevitably leads to depolarization of myocyte RP due to the electrotonic interaction between these cells; moreover, it follows from Eq. 2 that RP depolarization in the myocyte increases with the growth of *n* and/or *ɡ_gap_*.

The relative depolarization of the RP in *model 2* compared to *model 1* also predetermines the difference between both calcium and force signals in these two models, shown in Figure 3, so that the calcium transient and force in *model 2* are lower than in *model 1*. Indeed, the depolarized RP in *model 2* reduces, in particular, the electrical gradient for the calcium ions carried by *i*_*CaL*_ and therefore reduces this current as such. Reducing the number of Ca^2+^ ions entering with this current during any twitch gradually (twitch-to-twitch) reduces the calcium load of the SR compared to *model 1*. Hence, the calcium concentration in the SR is lower in *model 2* than in *model 1* when the steady-state twitches in both models are compared. Thus, during the steady-state twitches, the number of Ca^2+^ ions entering the cytosol from both outside and SR in *model 2* turns out to be smaller than in *model 1*. This is the cause of the difference observed in Figure 3 between the calcium transients in these two models, which in turn underlies the difference between the respective forces.

It would be quite tempting to consider some calcium unloading of the SR (as observed in *model 2* during the interaction of cardiomyocyte with fibroblasts) as a mechanism that might counteract arrhythmogenic overload of the SR in the case of an increase in *i*_*CaL*_ conductance. Unfortunately, our results presented in Figures 7, 8 suggest that this counteraction fails to prevent such overload. Moreover, the relative depolarization of the resting potential in *model 2*, which underlies the unloading of the SR, on the other hand, is itself an arrhythmogenic factor. The specific contribution of this depolarization, which arises in *model 2* in response to the interaction between cardiomyocyte and fibroblasts, to the amplification of EADs shown in Figures 7, 8 is discussed below.

### 4.3 Increased *i*_*CaL*_ and proarrhythmic events

Note that the authors of the paper cited above (Sridhar et al., 2017), who used a mathematical model of the electrical interaction of fibroblasts with a cardiomyocyte under conditions of increased *i*_*CaL*_ in the cardiomyocyte, did obtain EADs in response to an increase in the number of fibroblasts, but only under an additional condition. Without considering this condition, triggered activity did not occur in their simulations as well. Specifically, they had to artificially increase depolarization of RP in the fibroblasts interacting electrically with the myocyte because such depolarization by itself in response to the interaction was too weak in the *MacCannell* model they used (MacCannell et al., 2007).

Of note, in the *TP + M* model of the cardiomyocyte used in our study to combine with fibroblast models, EADs do occur due to the cardiomyocyte overload with calcium caused by a sufficiently large increase in *i*_*CaL*_. For example, Figure 8A shows EADs in a simulated single myocyte at a twofold increase in *i*_*CaL*_ conductance compared to the norm. The mechanical activity of the cardiomyocyte together with MEF and MCF are fundamentally important factors for the occurrence of EADs in the *TP + M* model (as discussed a few paragraphs below). Therefore, in the original *TNNP* electrophysiological model, proarrhythmic events do not occur at all under the same conditions when simulating a cardiomyocyte that is not connected to fibroblasts (Sridhar et al., 2017). Below we will discuss the mechanism of proarrhythmic activity triggered by calcium overload of the cardiomyocyte in the *TP + M* model and the contribution of MCF to this mechanism. However, it should be emphasized that as *model 1* revealed, electrotonic interaction with fibroblasts was not enough as such to affect the arrhythmogenic response of the cardiomyocyte to its calcium overload by an increase in *i*_*CaL*_. Indeed, the equal levels of *i*_*CaL*_ conductance in the cardiomyocyte alone and in the same cardiomyocyte electrically connected to the fibroblasts either resulted in EADs in both cases or did not cause triggered activity in any one of them. Neither the number of fibroblasts nor gap junction conductance alter this complete coincidence of responses to increased calcium current in *model 1*.

The interplay of electrotonic and mechanical interactions between the cardiomyocyte and fibroblasts implemented in *model 2* showed a completely different pattern. Even with a relatively modest increase in *i*_*CaL*_ conductance (1.4-fold increase compared to the norm) EADs occur in the cardiomyocyte included in *model 2* together with four fibroblasts (*ɡ*_*gap*_ = 3.0 nS), whereas no proarrhythmic disturbances occur in the same virtual single cardiomyocyte (Figure 7). The twofold increase in *i*_*CaL*_ conductance compared to the norm causes the appearance of EADs already in the single cardiomyocyte. In *model 2*, such an increase in *i*_*CaL*_ in the same myocyte, even with only 2 fibroblasts attached, causes not only EADs but also early extrasystoles (Figure 8). As might be expected, both an increase in fibroblasts (from 2 to 4) and an increase in gap junction conductance (from 1.0 nS to 3.0 nS) increase the extrasystolic vulnerability of the myocyte (Figure 9).

Let us discuss the mechanisms underlying these events in an ensemble of cardiomyocyte and fibroblasts that are electrically and mechanically connected. These are, on the one hand, internal mechanisms in the myocyte that trigger arrhythmogenic events in it and, on the other hand, amplifying mechanisms related to the coupling of myocytes and fibroblasts.

### 4.4 Internal mechanisms in the myocyte

**FIGURE 11:**
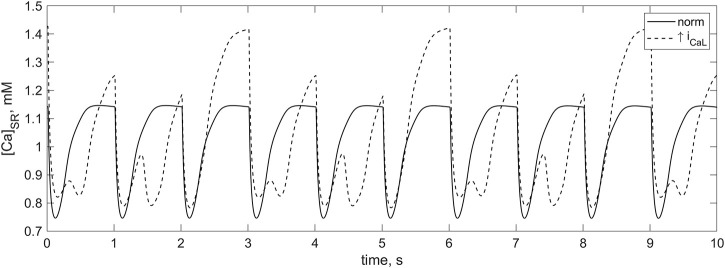
Calcium SR concentration in the *TP + M* model shown for 10 s. There is 1 Hz cardiomyocyte stimulation at **normal** *i*_*CaL*_ conductance (solid line) and at **twofold** increase in *i*_*CaL*_ conductance (dashed line). In the case of the increased *i*_*CaL*_ conductance at which the calcium overload of the myocyte occurs, the signals of greater and smaller amplitude alternate. When diastolic loading of the SR corresponds to signals of greater amplitude, spontaneous partial calcium release from the SR before the next stimulus occurs in addition to the regular calcium release. Due to this additional release, SR does not have time to fill to the same high level until the next stimulus.

An increase in *i*_*CaL*_ leads to an overload of the cardiomyocyte with calcium. Figure 11 shows an increase in Ca^2+^ level in sarcoplasmic reticulum (SR) in response to twofold increase in this current as compared to the normal *i*_*CaL*_. In both cases, the data are presented for the cardiomyocyte not associated with fibroblasts. The diastolic concentration of cytosolic Ca^2+^ and the peak Ca^2+^ transients also increase more than twofold. The overloading of the cardiomyocyte with Ca^2+^ in response to an increase in *i*_*CaL*_ is no less significant in the case when this cardiomyocyte is associated with fibroblasts. This overload is the main proarrhythmic factor in conditions of the increased *i*_*CaL*_. This factor is necessary for the occurrence of triggered activity, but is not always sufficient.

For example, no EADs arise in cardiomyocytes overloaded with calcium simulated in the electrophysiological *TNNP* model unless they interact with fibroblasts (Sridhar et al., 2017). We did obtain EADs even in the single cardiomyocyte applying the electromechanical *TP + M* model. The main difference between the *TP + M* model and the *TNNP* model used by Sridhar et al. is that the *TP + M* model accounts for cardiomyocyte mechanics, including MCF and MEF.

This difference suggests that mechanical feedbacks are to be responsible for the occurrence of EADs in the calcium-overloaded human myocyte. Detailed analysis of simulated EADs in the *TP + M* model shows that a feedback loop indeed plays a key role in these triggered activities, being based on the mechanism of cooperativity that makes CaTnC kinetics dependent on the number of attached force-generating Xbs. Overall, the observed arrhythmogenic disturbances arise due to the following sequence of intracellular processes in the calcium-overloaded cardiomyocyte, whether or not this myocyte is associated with fibroblasts.

**FIGURE 12:**
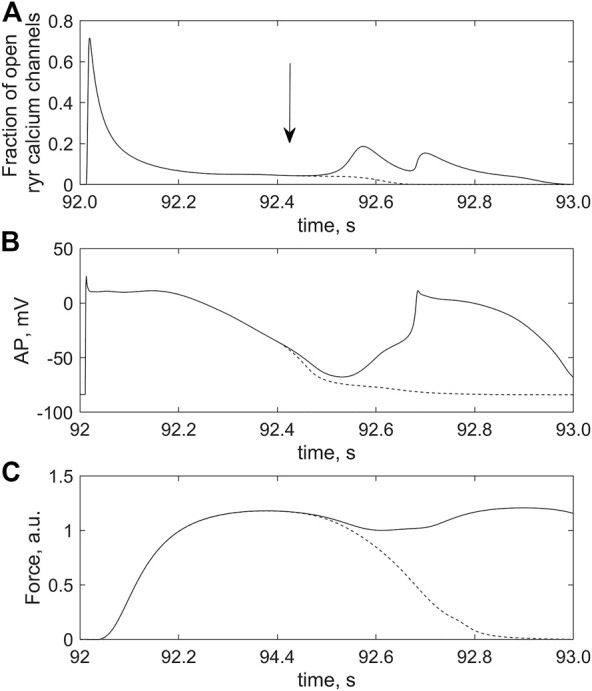
Effect of Xb-CaTnC cooperativity on the occurrence of triggered activity in the cardiomyocyte overloaded with calcium by the twofold increase in *i*_*CaL*_ conductance. The cardiomyocyte is coupled with 2 fibroblasts in *model 2*; *ɡ*_*gap*_ = 2.0 nS. **(A)** fraction of RyR calcium channels opened; **(B)** action potential (*AP*); **(C)** force generated (*Force*) after 96 s of regular stimulation with the rate of 1 Hz. The solid and dashed lines are used for two simulations (see text for details of the numerical experiments).

(1) During the myocyte relaxation phase, dissociation of CaTnC complexes increases due to the cooperative dependence of the CaTnC decay rate on the number of the attached Xbs. Ca^2+^ ions dissociated from TnC partially diffuse into the subspace surrounding the junctional SR.
(2) Under conditions of moderate overload of the myocyte with cytosolic and sarcoplasmic reticular Ca^2+^, the following process occurs: the Ca^2+^ ions rapidly dissociated from TnC maintain the excess of calcium level in the cytosol until the end of the refractoriness of the RyR channel, which arouse after the Ca^2+^ release induced by the previous regular stimulus. This refractoriness ends just during the relaxation phase. The term “excess level” in this context refers to such a level of cytosolic calcium that is sufficient for the Ca^2+^-induced additional opening of the RyR channels, i.e., for the initiation of spontaneous Ca^2+^-induced Ca^2+^ release from the SR into the cytosol between regular electrical stimuli (e.g., solid line, Figure 12A). We have previously referred to the sequence of events described in (1), 2 as the mechanism of “Xb-induced spontaneous Ca^2+^ release” (Sulman et al., 2008).
(3) Because of the same overload, the amount of calcium ions spontaneously released from SR is quite large to activate the Na^+^-Ca^2+^ current and thus contribute to the spontaneous voltage spike during or after repolarization of the cardiomyocyte AP.

Previously, we analyzed arrhythmic cellular dynamics triggered by Ca^2+^ overload in the framework of the *Ekaterinburg-Oxford* model describing excitation-contraction coupling in cardiomyocytes of laboratory animals (guinea pigs, rabbits) (Sulman et al., 2008). In the *Ekaterinburg-Oxford* model, we induced a gradual overloading of the myocyte with Ca^2+^ from beat to beat by partial inhibition of the membrane Na^+^-K^+^ ATPase and described the arrhythmogenic sequence of events (1)–(3) for the first time. The same sequence of events now occurs in the electromechanical model of the human cardiomyocyte, where overload is caused by increased *i*_*CaL*_. As a result, these events induce EADs in the simulated single human cardiomyocyte, whereas these proarrhythmic events may be amplified to early extrasystoles in the same myocyte interacting with fibroblasts. The contribution of the interaction between the myocyte and fibroblasts to such escalation of triggered activity in *model 2* is as follows.

### 4.5 Amplifying mechanisms associated with myocyte-fibroblast coupling

As shown above, when fibroblasts are coupled to a cardiomyocyte, the presence of mechanosensitive currents in fibroblasts mimicked in *model 2* significantly depolarizes RPs both in the fibroblasts and in the myocyte compared *to model 1*. This depolarization effect in *model 2* is length-dependent and increases significantly with the number of coupled fibroblasts as well as with the growth of *ɡ*_*gap*_. Thus, under conditions of Ca^2+^ overload of the cardiomyocyte interacting with fibroblasts, sufficiently large *n* and *ɡ*_*gap*_ promote the level of RP depolarization to reach the critical magnitude at which the following occurs. The spontaneous voltage spike (the last event in the sequence (1)–(3) above) reaches the level of AP, i.e., it initiates an extrasystole including an abnormal increase in myocyte active force (solid lines, Figures 12B,C).

Figure 9 shows combinations of *n* and *ɡ*_*gap*_ from the ranges 2 ≤ *n* ≤ 4 and 1.0 nS ≤ *ɡ*_*gap*_ ≤ 3 nS for which early extrasystoles occurred in *model 2*.

Note that when both the number of fibroblasts and gap junctional conductance are higher than the indicated ranges (*n* > 4 and *ɡ*_*gap*_ > 3.0 nS) proarrhythmic events are formally absent in *model 2*, but such a large *n* and *ɡ*_*gap*_ predetermine extreme cardiomyocyte failure in the model. Such cardiomyocytes are actually no longer able to contract, because so large number of fibroblasts and/or so high *ɡ*_*gap*_ conductance result in a strong drain of charge from the cardiomyocyte into the fibroblasts. This shortens APs and consequently the duration of *i*_*CaL*_ activated by the AP also decreases. Therefore, the amount of calcium entering the myocyte in response to each stimulus decreases (even despite the increased conductance of *i*_*CaL*_ itself), resulting in a gradual calcium unloading of the cardiomyocyte rather than in the overloading. As a result, arrhythmogenic overload with Са^2+^ does not occur in the cardiomyocyte. At the same time Ca^2+^ transients and, accordingly, the developed force become negligible.

An increase in dissociation of CaTnC complexes during the myocyte relaxation phase due to the cooperative dependence of CaTnC decay rate on the number of bound Xbs triggers the arrhythmogenic sequence of events (1)–(3) described above. Comparison of two simulations presented in Figure 12 provides direct evidence for the key role of the Xb-dependent rate of CaTnC decay in the occurrence of EADs. The solid lines in all panels of Figure 12 show time courses of the following variables of the cardiomyocyte coupled with two fibroblasts in *model 2* between two regular stimuli (at 92 s and at 93 s):

- time-varying fraction of the open RyR channels in the junctional SR (Figure 12A);
- action potential development (Figure 12B);
- cardiomyocyte force development (Figure 12C).

**FIGURE 13:**
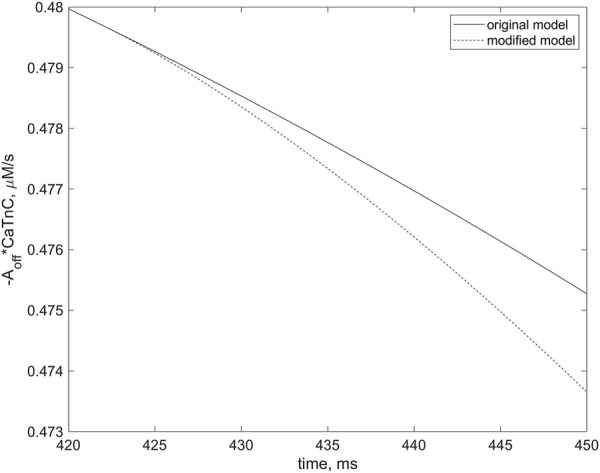
Differences in rates of the dissociation of CaTnC complexes in two simulations, indicating the role of cooperative mechanisms of this dissociation in EAD initiation. The curves correspond to the simulations depicted by dashed and solid lines, respectively, in Figure 12. The curve for the simulation in which the “constant” of [CaTnC] decay is willfully stopped in the numerical experiment to depend on the number of Xbs bound (dashed line) in contrast to the original simulation in which the EAD occurs (solid line). The dashed line slopes more steeply than the solid line. The left frame of the panel corresponds to the moment of the cooperativity dependence switch (420 ms), indicated by the arrow in Figure 12A. The curves are shown for the first 30 ms thereafter.

In response to the stimulus applied at 92 s, regular AP and contraction were followed by extrasystoles (Figures 12B, C). An important feature of this simulation was that the dissociation of the CaTnC complexes depended continuously and cooperatively on the instantaneous number of attached Xbs, in agreement with the main postulates of the *TP + M* model. In particular, the «constant» of the [CaTnC] decay during the relaxation phase increased all the time in response to the decreasing amount of Xbs. Hence, despite the significant decrease in the number of CaTnC complexes during the relaxation phase, the dissociation rate of these complexes (i.e., the rate of calcium release from TnС into the cytosol) almost did not decrease in that particular time interval in which the analyzed disturbance began to emerge (solid line, Figure 13). As a result, the excess of cytosolic calcium according to item (2) of the above sequence of events (1)–(3) triggered the arrhythmogenic additional opening of the RyR channels during cardiomyocyte relaxation (solid line, Figure 12A) and caused the extrasystole (solid lines, Figures 12B, C).

The second simulation, shown with the dashed lines in all panels of Figure 12, repeats the first simulation described above, shown with the solid lines, but only up to a point within the relaxation phase indicated by the vertical arrow in the top panel. This moment immediately precedes the extra increase in RyR channels opening during cardiomyocyte relaxation in the first simulation (solid line, Figure 12B). From the point in time marked by the arrow in this panel, the [CaTnC] decay “constant” stopped to depend continuously on the number of bound Xbs, as was in the case of the “solid” simulation, but was fixed at the value reached at this point in time for the further time interval. In other words, from the indicated time, the cooperative effect of Xbs kinetics on CaTnC dissociation was eliminated in the model for the “dashed” simulation Therefore, the dissociation rate of the CaTnC complexes decreased significantly after the indicated time (dashed line, Figure 13), since from this moment the decay rate is defined only by decrease of [CaTnC]. The reduced CaTnC dissociation rate prevented the excess calcium in the cytosol and thus the additional opening of RyR channels triggered by cytosolic calcium between stimuli (dashed line, Figure 12A). As a result, there was neither an extra AP nor an additional increase in force (dashed lines, Figures 12B, C).

Let us compare the results of our modeling with the previously studied well-known mechanisms of spontaneous trigger activity in cardiomyocytes. Two types of this trigger activity are usually considered, which may serve as precursor for the occurrence of arrhythmias in the myocardium (András et al., 2021). The normal course of the action potential can be disturbed by the occurrence of abnormal depolarizing inward currents both during the plateau or repolarization phase (EADs) and during diastole (delayed afterdepolarizations - DADs). When the change in action potential reaches a threshold value, an extra action potential and extrasystolic contraction may develop, spreading abnormal excitation and deformation in the myocardial tissue. The most common trigger activities are observed in cardiomyocytes overloaded with calcium (Volders et al., 2000). Calcium overload may be caused by a pathological weakening of Na^+^-K^+^-ATPase, by an increase in late Na^+^ current or by an increase in *i*_*CaL*_ (Wasserstrom and Aistrup, 2005; Sipido, 2006; Benitah et al., 2010; Shryock, 2011). The spontaneous release of calcium from the overloaded SR enhances the Na^+^-Ca^2+^ exchange current, which is depolarizing inward one during AP phases 2-4 and thus triggers afterdepolarization. Under various pathological conditions characterized by a decrease in potassium currents or a relatively depolarized resting potential, the repolarization reserve in the cardiomyocyte as a whole weakens. In such a case, the disturbances (EADs/DADs/Extra APs) can even be caused by a lower depolarizing current than in other cases (András et al., 2021). Thus, RP and prolonged AP (as a result of decreased potassium currents) are factors that increase vulnerability to arrhythmia (Burg and Attali, 2021).

A repolarization reserve forming during the early phase of repolarization protects the cardiomyocyte to some extent from abnormal afterdepolarization during the early repolarization phase (Trenor et al., 2017). However, as emphasized in the cited article by Trenor et al. this reserve is quite limited and can be weakened depending on the intensity of the depolarizing currents, including the *i*_*CaL*_ current. As our simulations show, increasing this current by 1.8 times or more can completely eliminate the Notch and even cause a small bulge in its place. *A priori*, even a case of a pronounced EAD, rather than just a so small bulge, could not be excluded. However, according to the predictions of the model, the repolarization reserve still protects the cardiomyocyte in the early repolarization phase from the afterdepolarization that could occur directly in response to the increased *i*_*CaL*_ current.

Note that in the simulations with increased *i*_*CaL*_ (Figure 7B and all panels in Figure 8), other EADs nonetheless occur, but not in the early phase preceding the plateau. They do arise during the final phase of AP repolarization, when *i*_*CaL*_ is almost completely inactivated and therefore cannot directly contribute to these afterdepolarization events.

The latter means that the simulated increase in *i*_*CaL*_ can only indirectly cause the EADs observed in the model, by gradual moderate overloading of the SR with calcium. The mechanisms underlying this type of the afterdepolarization, including the effects of the cross-bridges on CaTnC dissociation during the myocyte relaxation phase, are discussed above.

Overall, the modeling presented suggests that not only various current abnormalities but also mechanical factors contribute significantly to the arrhythmogenicity in calcium-overloaded human cardiomyocytes, especially when they are electromechanically coupled with fibroblasts. MEFs and MCFs in both the cardiomyocytes (Xb-induced spontaneous Ca^2+^ release) and fibroblasts (mechanosensitive ion channels) play key roles in this phenomenon.

### 4.6 Sensitivity of the obtained results to the parameters potentially critical to arrhythmogenicity

Our simulations revealed a significant contribution of the newly included mechanosensitive current *i*_*MS*_ in the fibroblast model to the studied arising EADs and extrasystoles in the cardiomyocyte coupled with fibroblasts. We additionally verified the effects of varying of the current parameters on these arrhythmogenic manifestations. When varying the current parameters over a wide range (up to twice higher or lower than the baseline values), we obtained results qualitatively similar to those presented above. An increase in the mechanosensitive current raises cardiomyocyte and fibroblast RPs, increasing the arrhythmogenic vulnerability. For example, due to the twofold increase in the reversal potential of *i*_*MS*_, trigger activity in the coupled cardiomyocyte arose even when *i*_*CaL*_ was increased only 1.5-fold compared to the norm. The variations in *i*_*MS*_ parameters are described in more detail in the Supplementary Materials.

Furthermore, we verified sensitivity of the studied effects to changes in the model parameters of those ionic currents in the calcium-overloaded cardiomyocyte, which could potentially contribute to its arrhythmogenic vulnerability (additionally to the conductance of *i*_*CaL*_, which we have already varied and analyzed as such).

In particular, we varied the conductances of the potassium currents in the model, since under various pathological conditions characterized by a decrease in potassium currents, the repolarization reserve in the cardiomyocyte as a whole weakens, which increases the vulnerability to arrhythmias (Burg and Attali, 2021). We varied the parameters of the potassium currents over a wide range of values and found out that a decrease in *i*_*Kr*_ and *i*_*Ks*_ conductances in the simulated cardiomyocyte caused the trigger activity in the cardiomyocyte less calcium overloaded, i.e., for smaller increase in the *i*_*CaL*_ conductance. Specifically, in the case of the twofold reduced *i*_*Kr*_ and *i*_*Ks*_, EADs occurred in simulations with the 1.4-fold increase in the conductance of *i*_*CaL*_ and extra APs with the 1.5-fold increase (compared to the baseline).

There are experimental data indicating that the paracrine interaction between cardiomyocytes and fibroblasts leads by itself to a decrease not only in the expression of *i*_*Kr*_ channels but also in SERCA expression (Cartledge et al., 2015; Johnson et al., 2020). Therefore, we additionally verified the effect of SERCA pump rate on the specific arrhythmogenic vulnerability of the cardiomyocytes associated with calcium overload. A twofold decrease in the parameter responsible for the maximum calcium uptake rate by the SR in the model partially unloaded the cardiomyocyte, so that trigger activity only occurred with a 2.8-fold increase in the conductance of *i*_*CaL*_ or higher. The latter simulations give a reason to suppose that paracrine interaction between fibroblasts and cardiomyocytes overloaded with calcium may influence the arrhythmogenic vulnerability in such myocytes in an ambiguous way: slowing *i*_*Kr*_ may increase this vulnerability, whereas slowing SERCA may decrease it.

## 5 Model limitations

The experiments presented by Abramochkin and co-authors (Abramochkin et al., 2014) identifying the current $i_{MS}$ through the mechanosensitive channels MSC-FB in fibroblasts were performed on rat cardiac fibroblasts, not on human ones. Therefore, the validation of the parameters of $i_{MS}$ as described in our model is a subject to certain conditions of uncertainty. For avoiding this lack, we have assessed the sensitivity of the obtained results to the variations of these parameters (see Section 4.6). It turns out that the main results are qualitatively independent of the specific values of the $i_{MS}$ parameters.

The description of MSC-FB as a non-selective cation channel could also be seen as a limitation of the developed *model 2.* Non-selectivity means that these channels transport cations of different types (predominantly Ca^2+^, Mg^2+^, Na^+^ and K^+^ (Stewart and Turner, 2021)) across the fibroblast membrane. As a result, the concentrations of these cations in the cell can actually change. Unfortunately, it is still unknown how to measure experimentally fractions of different cations carried by MSC-FB across the fibroblast membrane and consequently how to account for these fractions in the model. In various models of myocardium electrical activity, non-selective channels are described simply as carriers of positive charges (Healy and McCulloch, 2005; Salvador et al., 2022). We also use this approach for the MSC-FB in our work. This simplified approach, despite its obvious shortcomings, seems appropriate in the absence of experimental data. On the other hand, the simplified non-specific description allowed us to focus on the mechanodependence of these channels and to evaluate how this mechanodependence as such revealed itself in the model.

Since trigger activity in our study is tightly related to calcium dynamics in the cardiomyocyte, other calcium-dependent mechanisms might modulate the results obtained in the model, i.e., the occurrence of EADs and extrasystoles. In particular, intercellular communication between cardiomyocytes and fibroblasts *via* gap junctions can be affected by changes in intracellular calcium concentration in the myocyte, as certain connexin isoforms are calcium-dependent. An increase in intracellular calcium could hypothetically result in an inhibition of electrical coupling between the cells, although the way calcium influences the conductance of specific connexin isoforms and the exact level of calcium concentration required for an effective influence have not yet been sufficiently studied (Decrock et al., 2011; Rodríguez-Sinovas et al., 2021). Based on our results, we can predict the following effects of the calcium dependence of gap junction conductance. If the change in intracellular calcium concentration during cardiomyocyte overload is sufficient to inhibit partially the current through the gap junction, then this could attenuate the proarrhythmic effect of the interaction between fibroblasts and myocytes (see the table in Figure 9). However, this does not exclude the occurrence of trigger activity in calcium-overloaded cardiomyocytes, as EADs were also observed in our simulations even in the single cardiomyocytes in the case of its higher calcium overload (Figure 8A). The exact effects of calcium dependence of gap junction are still unclear, and mathematical modeling is hardly capable to clarify them as long as this calcium dependence is so poorly studied.

Potassium currents are known to contribute to proarrhythmic AP prolongations under various pathological conditions (Burg and Attali, 2021). Experimental findings show that both inward rectifier K^+^ current (*i*_*K1*_) and delayed rectifier K^+^ current (*i*_*Ks*_) in cardiomyocytes are likely to be regulated by intracellular calcium, which is not yet taken into account in our model. Bartos et al. showed that elevation of intracellular calcium concentration results in the enhancing of *i*_*Ks*_ amplitude and a negative shift in the voltage dependence of its activation in rabbit ventricular myocytes (Bartos et al., 2017). They also showed that *i*_*Ks*_ is usually maximally activated by calcium at [Ca^2+^]_i_ > 600 nM, i.e., under conditions of normal calcium transient dynamics, and further increase in [Ca^2+^]_i_ does not affect *i*_*Ks*_ activity. Therefore, the effect of the calcium dependence of *i*_*Ks*_ will manifest itself only under conditions of low intracellular calcium, which does not correspond to the calcium overload of cardiomyocytes considered in our work. However, when using our model to study other pathological conditions (e.g., heart failure) in which cardiomyocyte calcium decreases, the calcium dependence of *i*_*Ks*_ should be taken into account. A reduction in repolarizing *i*_*Ks*_ in such pathologies could prolong AP and increase susceptibility to triggering events in cardiomyocytes. The experimental data on the calcium dependence of *i*_*K1*_ are more controversial due to the different experimental conditions. Investigation of the effects of a non-adrenergic rise in [Ca^2+^]_i_ on the amplitude of *i*_*K1*_ in canine and human ventricular myocardium has shown augmentation of *i*_*K1*_ and AP shortening (Nagy et al., 2013). The authors concluded that these effects are mediated in part by a CaMKII-dependent pathway and may facilitate the repolarization reserve and protect the cardiomyocyte from trigger events. However, inhibition of *i*_*K1*_ by an increase in intracellular calcium is observed in other experimental studies (Mazzanti and DeFelice, 1990; Fauconnier et al., 2005).

This decrease in outward current may lead to a prolongation of AP in cardiomyocytes and contribute to an increased vulnerability of the myocardium to arrhythmias. These experimental controversies do not allow us to include the calcium dependence of *i*_*K1*_ in the model, despite its potential importance.

In conclusion, the repolarization reserve in the cardiomyocyte, which protects it from the abnormal trigger activity, is controlled by many mechanisms and signaling pathways. In our work, we have specifically focused on the effects of the electromechanical interaction between fibroblasts and cardiomyocytes, which under pathological conditions of calcium overload of the cardiomyocyte can reduce the repolarization reserve of the myocyte and thus facilitate its trigger activity.

## Data Availability

The original contributions presented in the study are included in the article/Supplementary Material, further inquiries can be directed to the corresponding author.
