# Supplementary material for: In silico analysis of the contribution of cardiomyocyte-fibroblast electromechanical interaction to the arrhythmia
Source: Front Physiol. 2023 Mar 10;14:1123609. doi: 10.3389/fphys.2023.1123609 (PMC10036780; doi:10.3389/fphys.2023.1123609)
Supplement: Supplementary file 1 [file DataSheet1.PDF]

I. TP+M model

State variables

	Definition	Initial value	Unit
V	membrane potential	-85.86	mV
d	voltage-dependent activation gate (i_{CaL})	$3.102 \cdot 10^{-5}$	dimensionless
f_2	fast voltage-dependent inactivation gate (i_{CaL})	0.9995	dimensionless
$fCass$	intracellular Ca^{2+} inactivation gate (i_{CaL})	1.0	dimensionless
f	slow voltage-dependent inactivation gate (i_{CaL})	0.98	dimensionless
R	proportion of closed I_{rel} channels	0.9876	dimensionless
O	proportion of opened I_{rel} channels	0.0	dimensionless
I	proportion of inactivated I_{rel} channels	0.0	dimensionless
RI	proportion of resting inactivated I_{rel} channels	0.0124	dimensionless
Ca_{sr}	sarcoplasmic reticulum Ca^{2+} concentration	1.19	mM
Ca_{ss}	subspace Ca^{2+} concentration	0.00018	mM
$CaTnC$	Ca^{2+}-troponin C complexes concentration	0.00770	mM
h	fast inactivation gate (i_{Na})	0.7617	dimensionless
j	slow inactivation gate (i_{Na})	0.7602	dimensionless
m	activation gate (i_{Na})	0.0019	dimensionless
$Xr1$	activation gate (i_{Kr})	0.00019	dimensionless
$Xr2$	inactivation gate (i_{Kr})	0.4777	dimensionless
Xs	activation gate (i_{Ks})	0.0034	dimensionless
r	voltage-dependent activation gate (i_{to})	$2.1 \cdot 10^{-8}$	dimensionless
s	voltage-dependent inactivation gate (i_{to})	1.0	dimensionless
K_i	intracellular K^+ concentration	134.72	mM
Na_i	intracellular Na^+ concentration	11.31	mM
Ca_i	intracellular Ca^{2+} concentration	0.000052	mM
l_1	deformation of CE against its slack length	0.392	μm
l_2	deformation of PE against its slack length	0.392	μm
l_3	deformation of XSE against its slack length	0.052	μm
v	velocity of CE deformation	0	μm/ms
w	velocity of PE deformation	0	μm/ms
N	cross-bridges concentration	$4.58 \cdot 10^{-6}$	dimensionless

Constants

	Definition	Value	Unit
$stim_{amp}$	amplitude of i_{stim}	52	pA/pF
$stim_{dur}$	duration of i_{stim}	1	ms
$stim_{per}$	periodicity of i_{stim}	1000	ms
$stim_{start}$	start of i_{stim}	10	ms
F	Faraday constant	96485.3415	C/M
R	gas constant	8314.472	mJ·K^{-1}·M^{-1}

Constants (continued)

	Definition	Value	Unit
T	temperature	310	K
Cm	cell capacitance	0.185	μF
V_c	cytoplasmic volume	0.016404	mm^3
V_{sr}	sarcoplasmic reticulum volume	0.001094	mm^3
V_{ss}	subspace volume	0.0000547	mm^3
Ca_o	intracellular Ca^{2+} concentration	2	mM
K_o	extracellular K^+ concentration	5.4	mM
Na_o	extracellular Na^+ concentration	140	mM
g_{CaL}	maximal i_{CaL} conductance	0.00006	l/(F· s)
g_{bCa}	maximal i_{bCa} conductance	0.000592	nS/pF
K_{pCa}	Ca_i half-saturation constant of i_{pCa}	0.0005	mM
g_{pCa}	maximal i_{pCa} conductance	0.2476	pA/pF
Buf_{sr}	total sarcoplasmic buffer concentration	10	mM
Buf_{ss}	total subspace buffer concentration	0.4	mM
Buf_c	total (except $CaTnC$) cytoplasmic buffer concentration	0.13	mM
EC	Ca_{sr} half-saturation constant of k_{casr}	1.5	mM
$K_{buf_{sr}}$	Ca_{sr} half-saturation constant for sarcoplasmic buffer	0.3	mM
$K_{buf_{ss}}$	Ca_{SS} half-saturation constant for subspace buffer	0.00025	mM
K_{buf_c}	Ca_i half-saturation constant for cytoplasmic buffer	0.00085	mM
V_{leak}	maximal I_{leak} conductance	0.00036	ms^{-1}
V_{rel}	maximal I_{rel} conductance	0.1224	ms^{-1}
V_{xfer}	maximal I_{xfer} conductance	0.00456	ms^{-1}
K_{up}	half-saturation constant of I_{up}	0.00025	mM
V_{maxup}	maximal I_{up} conductance	0.00765	mM/ms
$k1_{prime}$	R to O and RI to I I_{rel} transition rate	2.1	$mM^{-2}\cdot ms^{-1}$
$k2_{prime}$	O to I and R to RI I_{rel} transition rate	0.025	$mM^{-1}\cdot ms^{-1}$
$k3$	O to I and R to RI I_{rel} transition rate	0.06	ms^{-1}
$k4$	I to O and RI to I I_{rel} transition rate	0.005	ms^{-1}
max_{sr}	maximum value of k_{casr}	2.5	dimensionless
min_{sr}	minimum value of k_{casr}	1	dimensionless
Π_{min}	parameter of Π_{N_A} function	0.02	dimensionless
s_c	parameter of $N_A(CaTnC, N)$ function	1.0	dimensionless
TnC_{tot}	total concentration of TnC	0.07	mM
k_A	cooperativity parameter	28.0	mM^{-1}
a_{off}	maximum rate constant for $CaTnC$ dissociation	0.17	ms^{-1}
a_{on}	rate constant for $CaTnC$ association	35.0	$mM^{-1}\cdot ms^{-1}$
g_{Na}	maximal i_{Na} conductance	14.838	nS/pF
g_{bna}	maximal i_{bNa} conductance	0.00029	nS/pF

Constants (continued)

	Definition	Value	Unit
g_{K1}	maximal i_{K1} conductance	5.405	nS/pF
g_{pK}	maximal i_{pK} conductance	0.0146	nS/pF
g_{Kr}	maximal i_{Kr} conductance	0.153	nS/pF
P_{kna}	relative i_{Ks} permeability to Na^+	0.03	dimensionless
g_{Ks}	maximal i_{Ks} conductance	0.392	nS/pF
g_{to}	maximal i_{to} conductance	0.735	nS/pF
K_{NaCa}	maximal i_{NaCa}	10000	pA/pF
K_{sat}	saturation factor for i_{NaCa}	0.1	dimensionless
Km_{Ca}	Ca_i half-saturation constant for i_{NaCa}	1.38	mM
Km_{Nai}	Na_i half-saturation constant for i_{NaCa}	87.5	mM
α	factor enhancing outward nature of i_{NaCa}	1	dimensionless
γ	voltage dependence parameter of i_{NaCa}	0.35	dimensionless
K_{mNa}	Na_i half-saturation constant for i_{NaK}	40	mM
K_{mK}	K_o half-saturation constant for i_{NaK}	1	mM
P_{NaK}	maximal i_{NaK}	2.724	pA/pF
λ	scale parameter of F_{CE}	250.0	AFU
α_1	exponential coefficient of F_{SE}	14.6	μm^{-1}
β_1	linear coefficient of F_{SE}	4.2	AFU
α_2	exponential coefficient of F_{PE}	14.6	μm^{-1}
β_2	linear coefficient of F_{PE}	0.009	AFU
α_3	exponential coefficient of F_{XSE}	55.0	μm^{-1}
β_3	linear coefficient of F_{XSE}	0.11	AFU
α_{vp_l}	exponential coefficient of F_{VS_1}	16.0	μm^{-1}
α_{vp_s}	exponential coefficient of F_{VS_1}	16.0	μm^{-1}
β_{vp_l}	linear coefficient of F_{VS_1}	0.1	AFU· ms/μm
β_{vp_s}	linear coefficient of F_{VS_1}	10	AFU· ms/μm
α_{vs_l}	exponential coefficient of F_{VS_2}	46.0	μm^{-1}
α_{vs_s}	exponential coefficient of F_{VS_2}	39.0	μm^{-1}
β_{vs_l}	linear coefficient of F_{VS_2}	20.0	AFU· ms/μm
β_{vs_s}	linear coefficient of F_{VS_2}	60.0	AFU· ms/μm
v_{max}	parameter of p function	0.0055	μm/ms
a	parameter of p function	0.25	dimensionless
d_h	parameter of P_{star} function	0.5	dimensionless
α_P	parameter of G_{star} function	4.0	dimensionless
α_G	parameter of G_{star} function	1.0	dimensionless
k_μ	parameter of M_A function	0.6	dimensionless
μ	parameter of M_A function	3.3	dimensionless
g_1	parameter of n_1 function	0.6	μm^{-1}
g_2	parameter of n_1 function	0.52	dimensionless
$n1_A$	parameter of n_1 function	0.5	dimensionless
$n1_B$	parameter of n_1 function	55	μm
$n1_C$	parameter of n_1 function	1	dimensionless
$n1_Q$	parameter of n_1 function	0.835	dimensionless
$n1_K$	parameter of n_1 function	1	dimensionless
$n1_\nu$	parameter of n_1 function	5	dimensionless

Constants (continued)

	Definition	Value	Unit
S_0	parameter of L_{oz} function	1.14	μm
S_{055}	parameter of L_{oz} function	0.55	μm
S_{046}	parameter of L_{oz} function	0.46	μm
κ_0	parameter of κ function	2.1	dimensionless
κ_1	parameter of κ function	0.55	dimensionless
κ_2	parameter of κ function	0.0	dimensionless
m_0	fraction of strongly attached Xb in steady state isometric conditions	0.9	dimensionless
q_1	parameter of q function	0.0173	ms^{-1}
q_2	parameter of q function	0.259	ms^{-1}
q_3	parameter of q function	0.0173	ms^{-1}
q_4	parameter of q function	0.015	ms^{-1}
α_Q	parameter of q function	10.0	dimensionless
β_Q	parameter of q function	5.0	dimensionless
x_{st}	parameter of q function	0.964285	dimensionless
$r0$	preload	2.552	AFU (for $L_{init} = 90\% L_{max}$)
F_{aft}	afterload	6.89	AFU (for $10\% F_{isom}$)
k_{phys_rel}	parameter of V_{phys_rel} function	0.05	ms^{-1}
a_{phys_rel}	parameter of V_{phys_rel} function	*calculated*	μm
t_{phys_rel}	parameter of V_{phys_rel} function	*calculated*	ms
per_{phys_rel}	parameter of V_{phys_rel} function	230	ms

i_{stim}, stimulating current.

Calcium currents:
i_{CaL}, L-type Ca^{2+} current;
i_{bCa}, background Ca^{2+} current.

Calcium translocations:
I_{rel}, Ca^{2+} release from the sarcoplasmic reticulum (SR) via ryanodine receptors to the subspace (SS);
I_{xfer}, Ca^{2+} diffusion from SS to the cytoplasm (C);
I_{leak}, a small Ca^{2+} leakage from the SR to the cytoplasm;
I_{up}, Ca^{2+} pumping from the cytoplasm to the SR.
O, open conducting state of I_{rel}; R, resting closed state of I_{rel}; I, inactivated closed state of I_{rel}; RI, resting inactivated closed state of I_{rel}.

Calcium buffers:
CaB, buffering by other than $CaTnC$ intracellular ligands;
$CaTnC$, Ca^{2+}-troponin C complexes complexes;
Π_{N_A}, dependence defining cooperativity of the contractile proteins;
N_A, average fraction of the attached cross-bridges per one $CaTnC$ complex;
$CaSRB$, calcium buffering in SR;
$CaSSB$, subspace calcium buffering.

Sodium currents:
i_{Na}, fast Na^+ current;
i_{bNa}, background Na^+ current.

Potassium currents:
i_{K1}, inward rectifier K^+ current;
i_{to}, transient outward current;
i_{Kr}, i_{Ks}, rapid and slow delayed rectifier current;
i_{pK}, plateau K^+ current.

Pumps and exchangers:
i_{pCa}, sarcolemmal Ca^{2+} pump current;
i_{NaCa}, $Na^{+}\check{\ }Ca^{2+}$ exchanger current;
i_{NaK}, $Na^{+}\check{\ }K^{+}$ pump current.

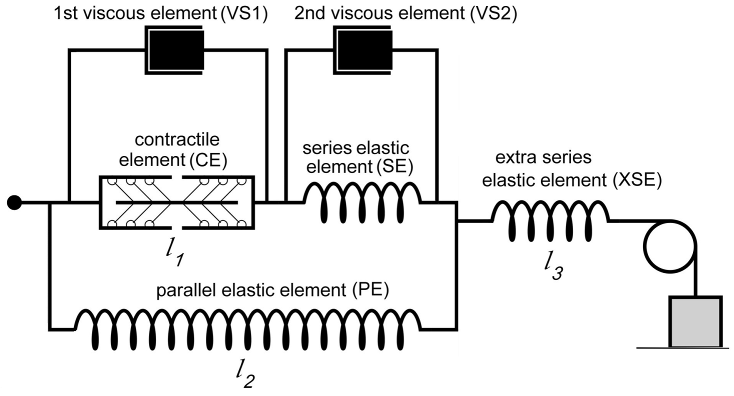

Figure 1: Rheological scheme in the TP+M model

Forces: AFU - arbitrary force unit;
F_{CE}, contractile element (sarcomere) (CE) force;
F_{SE}, serial elastic element (SE) force;
F_{PE}, parallel elastic element (PE) force;
F_{XSE}, extra serial elastic element (XSE) force;
F_{VS_1}, F_{VS_2}, viscous elements ($VS1$, $VS2$) forces.
l_1, deviation of contractile length from its slack length.
l_2, deviation of parallel elastic element length from its slack length.
l_3, deviation of extra series element length from its slack length.
$l = l_2 + l_3$, deviation of the sample length from its slack length.
p, dependence of the average cross-bridge force on the sarcomere shortening/lengthening velocity.
P_{star}, dependence of the steady-state sarcomere force on the sarcomere shortening/lengthening velocity.
G_{star}, dependence of the steady-state sarcomere stiffness on the velocity.
M_A, means end-to-end interaction between adjacent tropomyosin segments in the case if both of them affected by the respective $CaTnC$ complexes formation.

n_1, probability of that a myosin head can 'find' a vacant site on the actin filament.
L_{oz}, instantaneous length of thick and thin filament overlap zone.
κ, function required for variation the ratio between rates of cross-bridge attachment and detachment.
q, stationary relation 'stiffness-velocity' for the sample.
L_{init}, initial length of the sample.
L_{max}, corresponds to a sarcomere length equal to 2.23 μm.
F_{isom}, maximum of isometric force at given L_{init}.
V_{phys_rel}, dependence of sample length return during physiological relaxation.
k_{phys_rel}, physiological relaxation velocity.
a_{phys_rel}, physiological relaxation amplitude calculated as a difference between end-systolic and initial lengths of the sample.
t_{phys_rel}, time to start physiological relaxation fixed at the moment when $F_{sample} = r0$.
per_{phys_rel} period of physiological relaxation.

MODEL EQUATIONS

TNNP BLOCK (with modifications)

MEMBRANE POTENTIAL

$$i_{Stim} = \begin{cases} -stim_{amp} & \text{if } \left(time - \lfloor \frac{time}{stim_{per}} \rfloor \cdot stim_{per} \geq stim_{start}\right) \text{ and} \\ & \text{and } \left(time - \lfloor \frac{time}{stim_{per}} \rfloor \cdot stim_{per} \leq stim_{start} + stim_{dur}\right) \\ 0 & \text{otherwise} \end{cases}$$

$$\frac{\mathrm{d}V}{\mathrm{d}time} = \frac{-1}{Cm} \cdot (i_{K1} + i_{to} + i_{Kr} + i_{Ks} + i_{CaL} + i_{NaK} + i_{Na} + i_{b_{Na}} + i_{NaCa} + i_{b_{Ca}} + i_{p_K} + i_{p_{Ca}} + i_{Stim})$$

REVERSAL POTENTIALS

$$E_{Na} = \frac{R \cdot T}{F} \cdot \ln \frac{Na_o}{Na_i}$$

$$E_K = \frac{R \cdot T}{F} \cdot \ln \frac{K_o}{K_i}$$

$$E_{Ks} = \frac{R \cdot T}{F} \cdot \ln \frac{K_o + P_{kna} \cdot Na_o}{K_i + P_{kna} \cdot Na_i}$$

$$E_{Ca} = \frac{0.5 \cdot R \cdot T}{F} \cdot \ln \frac{Ca_o}{Ca_i}$$

L-type Ca^{2+} current

$$i_{CaL} = g_{CaL} \cdot d \cdot f \cdot f_2 \cdot f_{Cass} \cdot 4 \cdot \frac{(V-15) \cdot F^2}{R \cdot T} \cdot \frac{\left(0.25 \cdot Ca_{ss} \cdot e^{\frac{2 \cdot (V-15) \cdot F}{R \cdot T}} - Ca_o\right)}{e^{\frac{2 \cdot (V-15) \cdot F}{R \cdot T}} - 1}$$

L-type Ca^{2+} current. d gate

$$d_{inf} = \frac{1}{1+e^{\frac{-8-V}{7.5}}}$$

$$\alpha_d = \frac{1.4}{1+e^{\frac{-35-V}{13}}} + 0.25$$

$$\beta_d = \frac{1.4}{1+e^{\frac{V+5}{5}}}$$

$$\gamma_d = \frac{1}{1+e^{\frac{50-V}{20}}}$$

$$\tau_d = 1 \cdot \alpha_d \cdot \beta_d + \gamma_d$$

$$\frac{\mathrm{d}d}{\mathrm{d}time} = \frac{d_{inf} - d}{\tau_d}$$

L-type Ca^{2+} current. $f2$ gate

$$f2_{inf} = \frac{0.67}{1+e^{\frac{V+35}{7}}} + 0.33$$

$$\tau_{f2} = 562 \cdot e^{\frac{-(V+27)^2}{240}} + \frac{31}{1+e^{\frac{25-V}{10}}} + \frac{80}{1+e^{\frac{V+30}{10}}}$$

$$\frac{\mathrm{d}f_2}{\mathrm{d}time} = \frac{f2_{inf} - f2}{\tau_{f2}}$$

L-type Ca^{2+} current. fCa_{SS} gate

$$fCass_{inf} = \frac{0.6}{1+\left(\frac{Ca_{ss}}{0.05}\right)^2} + 0.4$$

$$\tau_{fCass} = \frac{80}{1+\left(\frac{Ca_{ss}}{0.05}\right)^2} + 2$$

L-type Ca^{2+} current. fCa_{SS} gate (continued)

$$\frac{\mathrm{d}f_{Cass}}{\mathrm{d}time} = \frac{fCass_{inf} - fCass}{\tau_{fCass}}$$

L-type Ca^{2+} current. f gate

$$f_{inf} = \frac{1}{1 + e^{\frac{V+20}{7}}}$$

$$\tau_f = 1102.5 \cdot e^{\frac{-(V+27)^2}{225}} + \frac{200}{1 + e^{\frac{13-V}{10}}} + \frac{180}{1 + e^{\frac{V+30}{10}}} + 20$$

$$\frac{\mathrm{d}f}{\mathrm{d}time} = \frac{f_{inf} - f}{\tau_f}$$

Ca^{2+} background current

$$i_{b_{Ca}} = g_{bca} \cdot (V - E_{Ca})$$

Ca^{2+} pump current

$$i_{p_{Ca}} = \frac{g_{pCa} \cdot Ca_i}{Ca_i + K_{pCa}}$$

Ca^{2+}-induced-Ca^{2+} release flow (i_{rel})

$$kcasr = max_{sr} - \frac{max_{sr} - min_{sr}}{1 + \left(\frac{EC}{Ca_{sr}}\right)^2}$$

$$k1 = \frac{k1_{prime}}{kcasr}$$

$$k2 = k2_{prime} \cdot kcasr$$

$$\frac{\mathrm{d}R}{\mathrm{d}time} = (k4 \cdot RI - k2 \cdot R \cdot Ca_{ss}) - (k1 \cdot R \cdot (Ca_{ss})^2 - k3 \cdot O)$$

$$\frac{\mathrm{d}O}{\mathrm{d}time} = (k1 \cdot R \cdot (Ca_{ss})^2 - k3 \cdot O) - (k2 \cdot O \cdot Ca_{ss} - k4 \cdot I)$$

$$\frac{\mathrm{d}I}{\mathrm{d}time} = (k2 \cdot O \cdot Ca_{ss} - k4 \cdot I) - (k3 \cdot I - k1 \cdot RI \cdot (Ca_{ss})^2)$$

$$\frac{\mathrm{d}RI}{\mathrm{d}time} = (k3 \cdot I - k1 \cdot RI \cdot (Ca_{ss})^2) - (k4 \cdot RI - k2 \cdot R \cdot Ca_{ss})$$

$$i_{rel} = V_{rel} \cdot O \cdot (Ca_{sr} - Ca_{ss})$$

Ca^{2+} DYNAMICS

$$i_{up} = \frac{Vmax_{up}}{1 + \frac{K_{up}^2}{Ca_i^2}}$$

$$i_{leak} = V_{leak} \cdot (Ca_{sr} - Ca_i)$$

$$i_{xfer} = V_{xfer} \cdot (Ca_{ss} - Ca_i)$$

$$B_{Cabufc} = \frac{1}{1 + \frac{Buf_c \cdot K_{buf_c}}{(Ca_i + K_{buf_c})^2}}$$

$$B_{Cabufsr} = \frac{1}{1 + \frac{Buf_{sr} \cdot K_{buf_{sr}}}{(Ca_{sr} + K_{buf_{sr}})^2}}$$

$$B_{Cabufss} = \frac{1}{1 + \frac{Buf_{ss} \cdot K_{buf_{ss}}}{(Ca_{ss} + K_{buf_{ss}})^2}}$$

$$N_A = \frac{TnC_{tot} \cdot N \cdot sc}{L_{oz} \cdot CaTnC}$$

$$\Pi_{N_A} = \begin{cases} 1 & \text{if } N_A \leq 0 \\ \Pi_{min}^{N_A} & \text{if } N_A \leq 1 \\ \Pi_{min} & \text{otherwise} \end{cases}$$

$$\frac{\mathrm{d}CaTnC}{\mathrm{d}time} = a_{on} \cdot (TnC_{tot} - CaTnC) \cdot Ca_i - a_{off} \cdot e^{-k_A \cdot CaTnC} \cdot \Pi_{N_A} \cdot CaTnC$$

$$\frac{\mathrm{d}Ca_{sr}}{\mathrm{d}time} = B_{Cabufsr} \cdot (i_{up} - (i_{rel} + i_{leak}))$$

$$\frac{\mathrm{d}Ca_{ss}}{\mathrm{d}time} = B_{Cabufss} \cdot \left(\frac{-1 \cdot i_{CaL} \cdot Cm}{2 \cdot 1 \cdot V_{ss} \cdot F} + \frac{i_{rel} \cdot V_{sr}}{V_{ss}} - \frac{i_{xfer} \cdot V_c}{V_{ss}} \right)$$

$$CaB = \frac{Buf_c \cdot Ca_i}{Ca_i + K_{buf_c}}$$

$$CaSRB = \frac{Buf_{sr} \cdot Ca_{sr}}{Ca_{sr} + K_{buf_{sr}}}$$

$$CaSSB = \frac{Buf_{ss} \cdot Ca_{ss}}{Ca_{ss} + K_{buf_{ss}}}$$

FAST Na^{+} CURRENT

$$i_{Na} = g_{Na} \cdot m^{3} \cdot h \cdot j \cdot (V - E_{Na})$$

FAST Na^{+} CURRENT. h GATE

$$h_{inf} = \frac{1}{\left(1 + e^{\frac{V+71.55}{7.43}}\right)^{2}}$$

$$\alpha_h = \begin{cases} 0.057 \cdot e^{\frac{-(V+80)}{6.8}} & \text{if } V < -40 \\ 0 & \text{otherwise} \end{cases}$$

$$\beta_h = \begin{cases} 2.7 \cdot e^{0.079 \cdot V} + 310000 \cdot e^{0.3485 \cdot V} & \text{if } V < -40 \\ \frac{0.77}{0.13 \cdot \left(1 + e^{\frac{V+10.66}{-11.1}}\right)} & \text{otherwise} \end{cases}$$

$$\tau_h = \frac{1}{\alpha_h + \beta_h}$$

$$\frac{\mathrm{d}h}{\mathrm{d}time} = \frac{h_{inf} - h}{\tau_h}$$

FAST Na^{+} CURRENT. j GATE

$$j_{inf} = \frac{1}{\left(1 + e^{\frac{V+71.55}{7.43}}\right)^{2}}$$

$$\alpha_j = \begin{cases} \frac{\left(-25428 \cdot e^{0.2444 \cdot V} - 6.948 \cdot 10^{-6} \cdot e^{-0.04391 \cdot V}\right) \cdot (V + 37.78)}{1 + e^{0.311 \cdot (V+79.23)}} & \text{if } V < -40 \\ 0 & \text{otherwise} \end{cases}$$

$$\beta_j = \begin{cases} \frac{0.02424 \cdot e^{-0.01052 \cdot V}}{1 + e^{-0.1378 \cdot (V+40.14)}} & \text{if } V < -40 \\ \frac{0.6 \cdot e^{0.057 \cdot V}}{1 + e^{-0.1 \cdot (V+32)}} & \text{otherwise} \end{cases}$$

$$\tau_j = \frac{1}{\alpha_j + \beta_j}$$

$$\frac{\mathrm{d}j}{\mathrm{d}time} = \frac{j_{inf} - j}{\tau_j}$$

FAST Na^+ CURRENT. m GATE

$$m_{inf} = \frac{1}{\left(1 + e^{\frac{-56.86 - V}{9.03}}\right)^2}$$

$$\alpha_m = \frac{1}{1 + e^{\frac{-60 - V}{5}}}$$

$$\beta_m = \frac{0.1}{1 + e^{\frac{V + 35}{5}}} + \frac{0.1}{1 + e^{\frac{V - 50}{200}}}$$

$$\tau_m = 1 \cdot \alpha_m \cdot \beta_m$$

$$\frac{\mathrm{d}m}{\mathrm{d}time} = \frac{m_{inf} - m}{\tau_m}$$

Na^+ BACKGROUND CURRENT

$$i_{b_{Na}} = g_{bna} \cdot (V - E_{Na})$$

INWARD RECTIFIER K^+ CURRENT

$$\alpha_{K1} = \frac{0.1}{1 + e^{0.06 \cdot (V - E_K - 200)}}$$

$$\beta_{K1} = \frac{3 \cdot e^{0.0002 \cdot (V - E_K + 100)} + e^{0.1 \cdot (V - E_K - 10)}}{1 + e^{-0.5 \cdot (V - E_K)}}$$

$$xK1_{inf} = \frac{\alpha_{K1}}{\alpha_{K1} + \beta_{K1}}$$

$$i_{K1} = g_{K1} \cdot xK1_{inf} \cdot \sqrt{\frac{K_o}{5.4}} \cdot (V - E_K)$$

K^+ PLATEAU CURRENT

$$i_{p_K} = \frac{g_{pK} \cdot (V - E_K)}{1 + e^{\frac{25 - V}{5.98}}}$$

RAPID TIME DEPENDENT K^+ CURRENT

$$i_{Kr} = g_{Kr} \cdot \sqrt{\frac{K_o}{5.4}} \cdot Xr1 \cdot Xr2 \cdot (V - E_K)$$

RAPID TIME DEPENDENT K^+ CURRENT. XR1 GATE

$$xr1_{inf} = \frac{1}{1 + e^{\frac{-26-V}{7}}}$$

$$\alpha_{xr1} = \frac{450}{1 + e^{\frac{-45-V}{10}}}$$

$$\beta_{xr1} = \frac{6}{1 + e^{\frac{V+30}{11.5}}}$$

$$\tau_{xr1} = 1 \cdot \alpha_{xr1} \cdot \beta_{xr1}$$

$$\frac{\mathrm{d}Xr1}{\mathrm{d}time} = \frac{xr1_{inf} - Xr1}{\tau_{xr1}}$$

RAPID TIME DEPENDENT K^+ CURRENT. XR2 GATE

$$xr2_{inf} = \frac{1}{1 + e^{\frac{V+88}{24}}}$$

$$\alpha_{xr2} = \frac{3}{1 + e^{\frac{-60-V}{20}}}$$

$$\beta_{xr2} = \frac{1.12}{1 + e^{\frac{V-60}{20}}}$$

$$\tau_{xr2} = 1 \cdot \alpha_{xr2} \cdot \beta_{xr2}$$

$$\frac{\mathrm{d}Xr2}{\mathrm{d}time} = \frac{xr2_{inf} - Xr2}{\tau_{xr2}}$$

SLOW TIME DEPENDENT K^+ CURRENT

$$i_{Ks} = g_{Ks} \cdot Xs^2 \cdot (V - E_{Ks})$$

SLOW TIME DEPENDENT K^+ CURRENT. XS GATE

$$xs_{inf} = \frac{1}{1 + e^{\frac{-5-V}{14}}}$$

$$\alpha_{xs} = \frac{1400}{\sqrt{1 + e^{\frac{5-V}{6}}}}$$

SLOW TIME DEPENDENT K^+ CURRENT. XS GATE (CONTINUED)

$$\beta_{xs} = \frac{1}{1 + e^{\frac{V-35}{15}}}$$

$$\tau_{xs} = 1 \cdot \alpha_{xs} \cdot \beta_{xs} + 80$$

$$\frac{\mathrm{d}Xs}{\mathrm{d}time} = \frac{xs_{inf} - Xs}{\tau_{xs}}$$

TRANSIENT OUTWARD K^+ CURRENT

$$i_{to} = g_{to} \cdot r \cdot s \cdot (V - E_K)$$

TRANSIENT OUTWARD K^+ CURRENT. R GATE

$$r_{inf} = \frac{1}{1 + e^{\frac{20-V}{6}}}$$

$$\tau_r = 9.5 \cdot e^{\frac{-(V+40)^2}{1800}} + 0.8$$

$$\frac{\mathrm{d}r}{\mathrm{d}time} = \frac{r_{inf} - r}{\tau_r}$$

TRANSIENT OUTWARD K^+ CURRENT. S GATE

$$s_{inf} = \frac{1}{1 + e^{\frac{V+20}{5}}}$$

$$\tau_s = 85 \cdot e^{\frac{-(V+45)^2}{320}} + \frac{5}{1 + e^{\frac{V-20}{5}}} + 3$$

$$\frac{\mathrm{d}s}{\mathrm{d}time} = \frac{s_{inf} - s}{\tau_s}$$

Na^+-Ca^{2+} EXCHANGER CURRENT

$$i_{NaCa} = \frac{K_{NaCa} \cdot \left(e^{\frac{\gamma \cdot V \cdot F}{R \cdot T}} \cdot Na_i^3 \cdot Ca_o - e^{\frac{(\gamma-1) \cdot V \cdot F}{R \cdot T}} \cdot Na_o^3 \cdot Ca_i \cdot \alpha\right)}{\left(Km_{Nai}^3 + Na_o^3\right) \cdot (Km_{Ca} + Ca_o) \cdot \left(1 + K_{sat} \cdot e^{\frac{(\gamma-1) \cdot V \cdot F}{R \cdot T}}\right)}$$

Na^{+}-K^{+} PUMP CURRENT

$$i_{NaK} = P_{NaK} \cdot \frac{K_o \cdot Na_i}{(K_o + K_{mk}) \cdot (Na_i + K_{mNa}) \cdot (1 + 0.1245 \cdot e^{\frac{-0.1 \cdot V \cdot F}{R \cdot T}} + 0.0353 \cdot e^{\frac{-V \cdot F}{R \cdot T}})}$$

Ca^{2+} DYNAMICS

$$\frac{\mathrm{d}Ca_i}{\mathrm{d}t} = B_{Cabufc} \cdot \left(\frac{(i_{leak} - i_{up}) \cdot V_{sr}}{V_c} + i_{xfer} - \frac{(i_{b_{Ca}} + i_{p_{Ca}} - 2 \cdot i_{NaCa}) \cdot Cm}{V_c \cdot F} - \frac{\mathrm{d}CaTnC}{\mathrm{d}t} \right)$$

K^{+} DYNAMICS

$$\frac{\mathrm{d}K_i}{\mathrm{d}time} = \frac{-1 \cdot (i_{K1} + i_{to} + i_{Kr} + i_{Ks} + i_{pK} + i_{Stim} - 2 \cdot i_{NaK})}{1 \cdot V_c \cdot F} \cdot Cm$$

Na^{+} DYNAMICS

$$\frac{\mathrm{d}Na_i}{\mathrm{d}time} = \frac{-1 \cdot (i_{Na} + i_{b_{Na}} + 3 \cdot i_{NaK} + 3 \cdot i_{NaCa})}{1 \cdot V_c \cdot F} \cdot Cm$$

MECHANICAL BLOCK

FORCE

$$F_{CE} = \lambda \cdot p_v \cdot N$$

$$F_{SE} = \beta_1 \cdot \left(e^{\alpha_1 \cdot (l_2 - l_1)} - 1\right)$$

$$F_{PE} = \beta_2 \cdot \left(e^{\alpha_2 \cdot l_2} - 1\right)$$

$$F_{XSE} = \beta_3 \cdot \left(e^{\alpha_3 \cdot l_3} - 1\right)$$

$$F_{VS_1} = k_{P_{vis}} \cdot v$$

$$F_{VS_2} = k_{S_{vis}} \cdot (w - v)$$

$$F_{sample} = F_{XSE}$$

CONTRACTION MODES

$$contraction_{mode} = \begin{cases} isometry & \\ isotony & \text{if } (F_{sample} > F_{aft}) \text{ and } \big(l \le l_0 \cdot \big(1 + 1 \cdot 10^{-4}\big)\big) \\ isometric\_relaxation & \text{if } (l \ is \ end - systolic \ length) \text{ and } (F_{sample} > r0) \\ physiological\_relaxation & \text{starts after isometric relaxation when } (F_{sample} \le r0) \end{cases}$$

LENGTH

$$l = l_2 + l_3$$

$$\frac{\mathrm{d}l_1}{\mathrm{d}time} = v$$

$$\frac{\mathrm{d}l_2}{\mathrm{d}time} = w$$

$$V_{phys\_rel} = \frac{a_{phys\_rel} \cdot k_{phys\_rel} \cdot e^{(-k_{phys\_rel} \cdot (t - (t_{phys\_rel} + per_{phys\_rel}/2)))}}{(1 + e^{(-k_{phys\_rel} \cdot (t - (t_{phys\_rel} + per_{phys\_rel}/2)))})^2}$$

$$\frac{\mathrm{d}l_3}{\mathrm{d}time} = \begin{cases} -w & \text{if } contraction_{mode} = isometry \ and \ isometric\_relaxation \\ 0 & \text{if } contraction_{mode} = isotony \\ V_{phys\_rel} - w & \text{if } contraction_{mode} = physiological\_relaxation \end{cases}$$

$$alp_p = \begin{cases} \alpha_{vp_l} & \text{if } v \leq 0 \\ \alpha_{vp_s} & \text{otherwise} \end{cases}$$

$$k_{P_{vis}} = \begin{cases} \beta_{vp_l} \cdot e^{\alpha_{vp_l} \cdot l_1} & \text{if } v \leq 0 \\ \beta_{vp_s} \cdot e^{\alpha_{vp_s} \cdot l_1} & \text{otherwise} \end{cases}$$

$$\phi_\chi = \begin{cases} \frac{-\left(\lambda \cdot K_\kappa \cdot p_v + alp_p \cdot k_{P_{vis}} \cdot v^2 + \left(\alpha_2 \cdot \beta_2 \cdot e^{\alpha_2 \cdot l_2} + \alpha_3 \cdot \beta_3 \cdot e^{\alpha_3 \cdot l_3}\right) \cdot w\right)}{\lambda \cdot N \cdot p_{prime_v} + k_{P_{vis}}}, \\ \quad \text{if } contraction_{mode} = isometry\ and\ isometric\_relaxation \\ \frac{-\left(\lambda \cdot K_\kappa \cdot p_v + alp_p \cdot k_{P_{vis}} \cdot v^2 + \alpha_2 \cdot \beta_2 \cdot e^{\alpha_2 \cdot l_2} \cdot w\right)}{\lambda \cdot N \cdot p_{prime_v} + k_{P_{vis}}}, \\ \quad \text{if } contraction_{mode} = isotonic \\ \frac{-\left(\lambda \cdot K_\kappa \cdot p_v + alp_p \cdot k_{P_{vis}} \cdot v^2 + \alpha_2 \cdot \beta_2 \cdot e^{\alpha_2 \cdot l_2} \cdot w - \alpha_3 \cdot \beta_3 \cdot e^{\alpha_3 \cdot l_3} \cdot \left(V_{phys\_rel} - w\right)\right)}{\lambda \cdot N \cdot p_{prime_v} + k_{P_{vis}}}, \\ \quad \text{if } contraction_{mode} = physiological\_relaxation \end{cases}$$

$$\frac{\mathrm{d}v}{\mathrm{d}time} = \phi_\chi$$

$$alp_s = \begin{cases} \alpha_{vs_l} & \text{if } w \leq v \\ \alpha_{vs_s} & \text{otherwise} \end{cases}$$

$$k_{S_{vis}} = \begin{cases} \beta_{vs_l} \cdot e^{\alpha_{vs_l} \cdot (l_2 - l_1)} & \text{if } w \leq v \\ \beta_{vs_s} \cdot e^{\alpha_{vs_s} \cdot (l_2 - l_1)} & \text{otherwise} \end{cases}$$

$$\frac{\mathrm{d}w}{\mathrm{d}time} = \begin{cases} \phi_\chi - alp_s \cdot (w-v)^2 - \dfrac{\alpha_1 \cdot \beta_1 \cdot e^{\alpha_1 \cdot (l_2 - l_1)} \cdot (w-v) + \left(\alpha_2 \cdot \beta_2 \cdot e^{\alpha_2 \cdot l_2} + \alpha_3 \cdot \beta_3 \cdot e^{\alpha_3 \cdot l_3}\right) \cdot w}{k_{S_{vis}}}, \\ \text{if } (contraction_{mode} = isometry\ and\ isometric\_relaxation) \\ \dfrac{k_{S_{vis}} \cdot \left(\phi_\chi - alp_s \cdot (w-v)^2\right) - \alpha_1 \cdot \beta_1 \cdot e^{\alpha_1 \cdot (l_2 - l_1)} \cdot (w-v) - \alpha_2 \cdot \beta_2 \cdot e^{\alpha_2 \cdot l_2} \cdot w}{k_{S_{vis}}}, \\ \text{if } (contraction_{mode} = isotony) \\ \phi_\chi - alp_s \cdot (w-v)^2 - \\ -\dfrac{\alpha_1 \cdot \beta_1 \cdot e^{\alpha_1 \cdot (l_2 - l_1)} \cdot (w-v) + \alpha_2 \cdot \beta_2 \cdot e^{\alpha_2 \cdot l_2} \cdot w - \alpha_3 \cdot \beta_3 \cdot e^{\alpha_3 \cdot l_3} \cdot (V_{phys\_rel} - w)}{k_{S_{vis}}}, \\ \text{if } (contraction_{mode} = physiological\_relaxation) \end{cases}$$

$$v_1 = \frac{v_{max}}{10}$$

$$\gamma_2 = \frac{a \cdot d_h \cdot \left(\frac{v_1}{v_{max}}\right)^2}{3 \cdot a \cdot d_h - \frac{(a+1) \cdot v_1}{v_{max}}}$$

$$P_{star} = \begin{cases} \frac{a \cdot \left(1 + \frac{v}{v_{max}}\right)}{a - \frac{v}{v_{max}}} & \text{if } v \leq 0 \\ 1 + d_h - \frac{d_h^2 \cdot a}{\frac{a \cdot d_h}{\gamma_2} \cdot \left(\frac{v}{v_{max}}\right)^2 + \frac{(a+1) \cdot v}{v_{max}} + a \cdot d_h} & \text{otherwise} \end{cases}$$

$$G_{star} = \begin{cases} 1 + \frac{0.6 \cdot v}{v_{max}} & \text{if } (v \leq 0) \\ \frac{P_{star}}{\frac{(0.4 \cdot a + 1) \cdot v}{a \cdot v_{max}} + 1} & \text{if } (0 < v) \text{ and } (v \leq v_1) \\ \frac{P_{star} \cdot e^{-\alpha_G \cdot \left(\frac{v - v_1}{v_{max}}\right)^{\alpha_P}}}{\frac{(0.4 \cdot a + 1) \cdot v}{a \cdot v_{max}} + 1} & \text{otherwise} \end{cases}$$

$$case_1 = \frac{a \cdot (0.4 + 0.4 \cdot a)}{v_{max} \cdot ((a+1) \cdot 0.4)^2}$$

$$case_2 = \frac{a \cdot 1 \cdot \left(1 + 0.4 \cdot a + \frac{1.2 \cdot v}{v_{max}} + 0.6 \cdot \left(\frac{v}{v_{max}}\right)^2\right)}{v_{max} \cdot \left(\left(a - \frac{v}{v_{max}}\right) \cdot \left(1 + \frac{0.6 \cdot v}{v_{max}}\right)\right)^2}$$

$$case_3 = \frac{0.4 \cdot a + 1}{a \cdot v_{max}}$$

$$case_4 = \frac{1}{v_{max}} \cdot e^{-\alpha_G \cdot \left(\frac{v - v_1}{v_{max}}\right)^{\alpha_P}} \cdot \left(\frac{0.4 \cdot a + 1}{a} + \alpha_G \cdot \alpha_P \cdot \left(1 + \frac{(0.4 \cdot a + 1) \cdot v}{a \cdot v_{max}}\right) \cdot \left(\frac{v - v_1}{v_{max}}\right)^{\alpha_P - 1}\right)$$

$$p_{prime_v} = \begin{cases} case_1 & \text{if } v \leq -v_{max} \\ case_2 & \text{if } (-v_{max} < v) \text{ and } (v \leq 0) \\ case_3 & \text{if } (0 < v) \text{ and } (v \leq v_1) \\ case_4 & \text{otherwise} \end{cases}$$

$$p_v = \frac{P_{star}}{G_{star}}$$

$$M_A = \frac{\left(\frac{CaTnC}{TnC_{tot}}\right)^{\mu} \cdot \left(1 + k_{\mu}^{\mu}\right)}{\left(\frac{CaTnC}{TnC_{tot}}\right)^{\mu} + k_{\mu}^{\mu}}$$

$$temp_{n1} = (g_1 \cdot l_1 + g_2) \cdot \left(n1_A + \frac{n1_K - n1_A}{\left(n1_C + n1_Q \cdot e^{-n1_B \cdot l_1}\right)^{\frac{1}{n1_{\nu}}}}\right)$$

$$n_1 = \begin{cases} 0 & \text{if } temp_{n1} < 0 \\ temp_{n1} & \text{if } temp_{n1} < 1 \\ 1 & \text{otherwise} \end{cases}$$

$$L_{oz} = \begin{cases} \frac{l_1 + S_0}{S_{046} + S_0} & \text{if } l_1 \leq S_{055} \\ \frac{S_0 + S_{055}}{S_{046} + S_0} & \text{otherwise} \end{cases}$$

$$\kappa = \begin{cases} \kappa_1 + \kappa_2 \cdot \frac{v}{v_{max}} & \text{if } v < 0 \\ \kappa_1 & \text{otherwise} \end{cases}$$

$$v_{st} = x_{st} \cdot v_{max}$$

$$q_v = \begin{cases} q_1 - \frac{q_2 \cdot v}{v_{max}} & \text{if } v \leq 0 \\ \frac{(q_4 - q_3) \cdot v}{v_{st}} + q_3 & \text{if } (v \leq v_{st}) \text{ and } (0 < v) \\ \frac{q_4}{\left(1 + \frac{\beta_Q \cdot (v - v_{st})}{v_{max}}\right)^{\alpha_Q}} & \text{otherwise} \end{cases}$$

$$k_{p_v} = \kappa \cdot \kappa_0 \cdot q_v \cdot m_0 \cdot G_{star}$$

$$k_{m_v} = \kappa_0 \cdot q_v \cdot (1 - \kappa \cdot m_0 \cdot G_{star})$$

$$K_{\kappa} = k_{p_v} \cdot M_A \cdot n_1 \cdot L_{oz} \cdot (1 - N) - k_{m_v} \cdot N$$

$$\frac{\mathrm{d}N}{\mathrm{d}time} = K_{\kappa}$$

II. EQUATIONS FOR FIBROBLASTS – MYOCYTE ELECTROTONIC INTERACTION inherited from the MacCannell2007 MODEL

For the modeling of the fibroblast-cardiomyocyte electrical interaction we used the MacCannell2007 model
(MacCannell KA, Bazzazi H, Chilton L, Shibukawa Y, Clark RB, Giles WR. A mathematical model of electrotonic interactions between ventricular myocytes and fibroblasts. Biophys J. 2007;92(11):4121-4132. doi:10.1529/biophysj.106.101410)

State variables

	Definition	Initial value	Unit
V_{cfi}	membrane potential across the i-th coupled fibroblast	-84.79	mV
Na_{i_f}	intracellular Na^+ concentration	10.91	mM
r_{Kv}	activation parameter	0.0	dimensionless
s_{Kv}	inactivation parameter	1.0	dimensionless

Constants

	Definition	Value	Unit
g_{gap}	fibroblast-to-myocyte conductance	between 0.5 and 3	nS
n	number of fibroblasts	between 0 and 3	dimensionless
C_{mf}	fibroblast membrane capacitance	6.3	pF
E_{k_f}	reversal potential	-87.0	mV
g_{Kv_f}	maximal i_{kv_f} conductance	0.25	nS/pF
g_{K1_f}	maximal i_{K1_f} conductance	0.4822	nS/pF
$i_{NaK_f max}$	maximal $i_{NaK_f max}$ current	2.002	pA/pF
K_{mK_f}	K_{o_f} half-saturation constant for i_{NaK_f}	1.0	mM/L
K_{mNa_f}	Na_{i_f} half-saturation constant for i_{NaK_f}	11.0	mM/L
V_{rev_f}	reversal potential of pump	-150.0	mV
B_f	empirically determined constant	-200.0	mV
g_{bNa_f}	maximal i_{bNa_f} conductance	0.0095	nS/pF
V_{c_f}	maximal i_{bNa_f} conductance	0.016404	nS/pF

CARDIOMYOCYTE MEMBRANE POTENTIAL

$$\frac{\mathrm{d}V}{\mathrm{d}time} = -\frac{1}{C_m} \cdot \Big(i_{K1} + i_{to} + i_{Kr} + i_{Ks} + i_{CaL} + i_{NaK} + i_{Na} + i_{b_{Na}}$$

$$+ \mathrm{i}_{NaCa} + i_{b_{Ca}} + i_{p_K} + i_{p_{Ca}} + i_{Stim} + \textstyle\sum_{i=1}^{n} g_{gap} \cdot (V - V_{cfi}) \Big)$$

MEMBRANE POTENTIAL IN THE I-TH FIBROBLAST

$$\frac{\mathrm{d}V_{cfi}}{\mathrm{d}time} = -\frac{1}{C_{mf}} \cdot \Big(i_{kv_f} + i_{K1_f} + i_{NaK_f} + i_{bNa_f} + g_{gap} \left(V_{cfi} - V\right) \Big)$$

TIME- AND VOLTAGE-DEPENDENT K^+ CURRENT IN THE I-TH FIBROBLAST

$$i_{kv_f} = g_{Kv_f} \cdot r_{Kv_f} \cdot s_{Kv_f} \cdot \left(V_{cfi} - E_{k_f}\right)$$

$$\frac{\mathrm{d}r_{Kv_f}}{\mathrm{d}time} = \frac{r_{Kv_f} - r_f}{\tau_{r_f}}$$

$$\frac{\mathrm{d}s_{Kv_f}}{\mathrm{d}time} = \frac{s_{Kv_f} - s_f}{\tau_{s_f}}$$

$$\tau_{r_f} = 20.3 + 138.0 \cdot e^{-\left(\frac{V_{cfi}+20.0}{25.9}\right)^2}$$

$$\tau_{s_f} = 1574.0 + 5268.0 \cdot e^{-\left(\frac{V_{cfi}+23.0}{22.7}\right)^2}$$

$$r_f = \frac{1}{1 + e^{-\frac{V_{cfi}+20.0}{11.0}}}$$

$$s_f = \frac{1}{1 + e^{-\frac{V_{cfi}+23.0}{7.0}}}$$

INWARD-RECTIFYING K^+ CURRENT IN THE I-TH FIBROBLAST

$$i_{K1_f} = g_{K1_f} \cdot \alpha_{K1_f} \cdot \frac{V_{cfi} - E_{k_f}}{\alpha_{K1_f} + \beta_{K1_f}}$$

$$\alpha_{K1_f} = \frac{0.1}{1 + e^{0.06 \cdot \left(V_{cfi} - E_{k_f} - 200.0\right)}}$$

INWARD-RECTIFYING K^+ CURRENT IN THE I-TH FIBROBLAST (CONTINUED)

$$\beta_{K1_f} = \frac{3.0 \cdot e^{0.0002 \cdot \left(V_{cfi} - E_{k_f} - 100.0\right)} + e^{0.1 \cdot \left(V_{cfi} - E_{k_f} - 10.0\right)}}{1 + e^{-0.5 \cdot \left(V_{cfi} - E_{k_f}\right)}}$$

$Na^+ - K^+$ PUMP CURRENT IN THE I-TH FIBROBLAST

$$i_{NaK_f} = i_{NaK_{fmax}} \cdot \frac{K_o}{K_o + K_{mK_f}} \cdot \frac{{Na_{i_f}}^{1.5}}{{Na_{i_f}}^{1.5} + K_{mNa_f}^{1.5}} \cdot \frac{V_{cfi} - V_{rev_f}}{V_{cfi} - B_f}$$

BACKGROUND Na^+ CURRENT IN THE I-TH FIBROBLAST

$$i_{bNa_f} = g_{bNa_f} \cdot \left(V_{cfi} - E_{Na_f}\right)$$

REVERSAL POTENTIAL

$$E_{Na_f} = \frac{R \cdot T}{F} \cdot \ln \frac{Na_o}{Na_{i_f}}$$

Na^+ DYNAMICS IN FIBROBLASTS

$$\frac{\mathrm{d}Na_{i_f}}{\mathrm{d}time} = -\frac{i_{bNa_f} + 3.0 \cdot i_{NaK_f}}{V_{c_f} \cdot F}$$

III. FIBROBLASTS – MYOCYTE ELECTROMECHANICAL INTERACTION

Based on the model of electrotonic interaction between cardiomyocytes and fibroblasts, a new model was constructed that also takes into account their mechanical interaction. We considered the following two factors of this interaction: 1) the existence of a passive elastic force of the fibroblast resulting from its deformation, and 2) mechanosensitive ion channels in fibroblasts detected in patch-clamp experiments. [Abramochkin et al, J Mol Cell Cardiol, 2014]. The conductance of mechanosensitive ion channels, as well as the elastic force, depends on the deformation of the fibroblast.

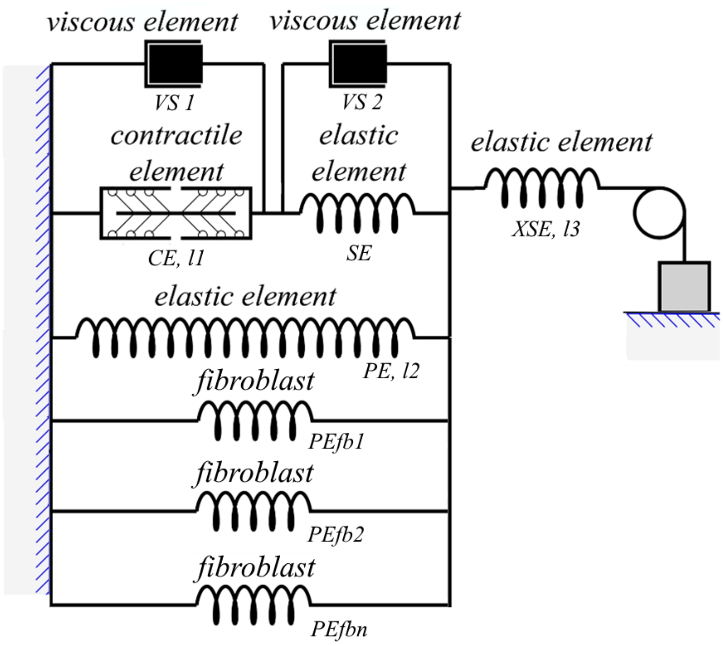

Figure 2: Rheological scheme of the model. Fibroblasts (PE_{fb1}, PE_{fb2},.. PE_{fbn}) are attached parallel to the elastic element (PE) of the cardiomyocyte, where n is the number of attached fibroblasts

Figure 2 shows a rheological scheme of the mechanical activity of cardiomyocyte and its mechanical interaction with fibroblasts. Fibroblasts are presented as passive elastic elements parallel to the elastic element of the cardiomyocyte. The force developed by the myocyte interacting with n attached fibroblasts is as follows:

$$F_{sample} = F_{XSE} = F_{PE} + n \cdot F_{PE_{fbn}} + F_{CE} + F_{VS1}$$
$$F_{PE_{fbn}} = \beta_{2_f} \cdot (e^{\alpha_{2_f} \cdot l_2} - 1),$$

where α_{2_f} and β_{2_f}, the exponential and linear coefficients of force $F_{PE_{fbn}}$, is set equal for all fibroblasts for simplicity. The force developed by the element PE_{fb2} depends on the deformation l_2 of the elastic element PE.

When modelling the current through the mechanosensitive channel in the fibroblast membrane, we relied on the I-V relationships presented for different deformation of fibroblast in Abramochkin et al. The compression and stretch of the fibroblast changed not only the slope of the I-V curves for the current but also the reverse potential. The mechanosensitivity of the current via fibroblast membrane i_{MS} can be written as follows:

$$i_{MS}(\Delta l, V_{cfi}) = g_{max} \cdot a(\Delta l) \cdot (V_{cfi} - V_{rev}(\Delta l)),$$

where Δl is the deformation of fibroblast during compression and stretch rel-

ative to its initial length, V_{cfi} is the fibroblast membrane potential, g_{max} is the i_{MS} conductance in control conditions, when fibroblast is not deformed, $a(\Delta l)$ is a deformation dependency factor of i_{MS}, ($a(\Delta l) = 1$ in control), V_{rev} is mechanodependent reversal potential.

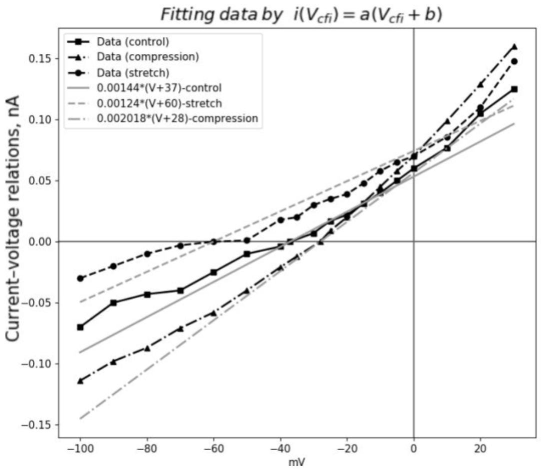

Figure 3: Approximation of experimental data from Abramochkin et al

Based on the rheological scheme, we correlated the relative deformation of fibroblasts and the deformation of cardiomyocytes. In our model, the slack length of the sarcomere is 1.67 μm. When the cardiomyocyte is prestretched to a length of $90\% L_{max}$, the deformation of the PE element is 0.385 μm, which corresponds to 23% of its slack length. Then, the relative deformation of the fibroblast during pre-stretch is also 23% of its slack length or $\Delta l_{stretch} = 0.23$. In the experiments of Abramochkin et al, the fibroblast was stretched and compressed by 2 μm relative to its initial length L_{fib}. In terms of relative deformation, the deformations of the fibroblast were $\Delta l_{comp} = 0.14$ after compression and $\Delta l_{stretch} = 0.32$ after stretching.

We approximated the experimental I-V curves of the Abramochkin group for the initial preload condition (control), compression, and stretch (Figure 3, black lines) by a linear dependence: $i_{MS}(V_{cfi}) = g_{max} \cdot a \cdot (V_{cfi} + b)$, where b - mechano-dependent reversal potential under control conditions, compression and stretch equal to -37 mV, -28 mV and -60 mV from experimental I-V curve; a - coefficient of mechano-dependence of slope of an approximate line.

Let's $a_{initial} = 1$, then $g_{max} = 0.00144$, $a_{stretch} = 0.860049$, $a_{comp} = 1.401134$. Then the linear functions approximating the experimental data have the form (represented by the gray lines in Figure 3):

$$control : i_{MS}(V_{cfi}) = g_{max} \cdot a \cdot (V_{cfi} + 37) = 0.00144 \cdot (V_{cfi} + 37),$$

$$stretch : i_{MS}(V_{cfi}) = g_{max} \cdot a_{stretch} \cdot (V_{cfi} + 60) = 0.00124 \cdot (V_{cfi} + 60),$$

$$compression : i_{MS}(V_{cfi}) = g_{max} \cdot a_{comp} \cdot (V_{cfi} + 28) = 0.002018 \cdot (V_{cfi} + 28).$$

Thus, we obtained three conductance values: $g_{max} \cdot a_{initial}$, $g_{max} \cdot a_{stretch}$, $g_{max} \cdot$

a_{comp}, which were approximated using experimental data for three different fibroblast lengths.
Let's put three points (Δl_{comp}, a_{comp}), ($\Delta l_{initial}$, $a_{initial}$), ($\Delta l_{stretch}$, $a_{stretch}$) in a diagram (Figure 4A). The previously calculated values of the relative deformation of the fibroblast lie on the abscissa axis (Δl_{comp}, $\Delta l_{initial}$, $\Delta l_{stretch}$) = (0.14, 0.23, 0.32). The corresponding coefficients of the mechano-dependence of the conductance of the mechanosensitive fibroblast current (a_{comp}, $a_{initial}$, $a_{stretch}$) = (1.401134, 0.00144, 0.860049) for compression (black dot), control (blue dot), and stretch (red dot) are on the ordinate axis. Similarly, in Figure 4B, the values of fibroblast deformation (Δl_{comp}, $\Delta l_{initial}$, $\Delta l_{stretch}$) = (0.14, 0.23, 0.32) and the values of reversal potential (V_{comp}, $V_{initial}$, $V_{stretch}$) = (-28 mV, -37 mV, -60 mV) are indicated.

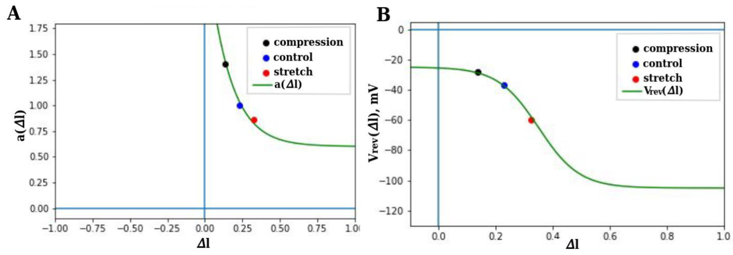

Figure 4: Dependencies of conductance parameter a (panel A) and reversal potential V_{rev} (panel B) on fibroblast deformation. Black dots represent the compression of the fibroblast; blue dots represent the original length of the fibroblast; red dots represent its elongation. Green lines are for approximate functions (Δl)

Based on the experimental values, we derived the dependence functions of the coefficient of mechano-dependence $a(\Delta l)$ (Figure 4A) and the reversal potential $V_{rev}(\Delta l)$ (Figure 4B) from the relative deformation of the fibroblast Δl:

$$a(\Delta l) = \frac{21}{1 + e^{7\cdot(\Delta l + 0.32)}} + 0.6,$$

$$V_{rev}(\Delta l) = \frac{80}{1 + e^{14\cdot(\Delta l - 0.35)}} - 105.$$

MECHANOSENSITIVE CURRENT FOR FIBROBLAST $i_{MS}(\Delta l, V_{cfi})$

$$\mathrm{i}_{MS}(\Delta l, V_{cfi}) = 0.00144 \cdot (\tfrac{21}{1+e^{7\cdot(\Delta l+0.32)}} + 0.6) \cdot (V_{cfi} - \tfrac{80}{1+e^{14\cdot(\Delta l-0.35)}} - 105).$$

MEMBRANE POTENTIAL IN THE I-TH FIBROBLAST

$$\frac{\mathrm{d}V_{cfi}}{\mathrm{d}time} = -\frac{1}{C_{mf}} \cdot \left[i_{kv_f} + i_{K1_f} + i_{NaK_f} + i_{bNa_f} + i_{MS}(\Delta l, V_{cfi}) + g_{gap}\,(V_{cfi} - V)\right]$$

The influence of the parameters of mechanosensitivity of the conductance of channel $a(\Delta l)$ and its reversal potential $V_{rev}(\Delta l)$, as well as the number of connected fibroblasts and the conductance of gap junctions g_{gap} (0.5 and 3 nS) on the electrical and mechanical functions of cardiomyocytes and on the membrane potential of fibroblasts was studied. The range of parameter variation was chosen so that the functions of the dependence of conductance and reverse potential on the relative deformation of the fibroblast (Δl) lie within an interval of 10% of the lines approximated by experimental data of Abramochkin et al.

Variation of the parameters $a(\Delta l)$ does not affect the electrical and mechanical

function of the cardiomyocyte either in the case of moderate gap junction conductance ($g_{gap} = 0.5$ nS), which corresponds to the physiological norm, or in the case of pathological gap junction conductance ($g_{gap} = 3.0$ nS). On the other hand, variations in the parameters of the reversal potential $V_{rev}(\Delta l)$ significantly affect the action potential and the force generated by the cardiomyocyte. By varying it, one can both increase and decrease the effect of current $i_{MS}(\Delta l, V_{cfi})$ on the membrane potential of fibroblasts and thereby on the resting potential, the duration of the action potential, and the force of the cardiomyocyte.

IV. COMPUTATIONAL METHODS

We implemented our model using Python on a PC with a 3.2 GHz Intel Core i7 processor with 32 Gb RAM. ODE systems were numerically solved using the SUNDIALS CVODE package (https://computing.llnl.gov/projects/sundials/cvode).

CVODE is a variable-order, variable-step multi-step algorithm for solving stiff and nonstiff ordinary differential equations.
